# Role of third molars in mandibular fractures: a systematic umbrella review with updated meta-analysis

**DOI:** 10.3389/froh.2026.1865229

**Published:** 2026-08-17

**Authors:** Shahen Hiwa Omer, Mohammed Khalid Mahmood, Mohammed Aso Abdulghafor, Yad Mariwan Mohammed Amin, Arthur Falguiere, Romain Lan

**Affiliations:** 1 Department of Dentistry, Komar University of Science and Technology, Sulaymaniyah, Iraq; 2 French National Center of Scientific Research (CNRS), French Blood Establishment (EFS), Bio-Cultural Anthropology, Law, Ethics and Health Laboratory (ADES), Faculty of Medical and Paramedical Sciences, Aix-Marseille University, Marseille, France; 3 College of Dentistry, Sulaymaniyah University, Sulaymaniyah, Iraq; 4 Department of Dentistry, Tishk International University, Sulaymaniyah, Iraq; 5 Oral Surgery Department, Timone University Hospital, Marseille, Provence-Alpes-Côte d'Azur, France

**Keywords:** angle fracture, condylar fracture, mandibular fracture, meta analysis, systematic review, third molar

## Abstract

**Background and aims:**

Previous meta-analyses (MAs) and systematic reviews (SRs) reported that presence of third molars increases the risk of angle and decreases the risk of condylar fractures, but the data is dispersed across these studies. The aim of this umbrella review is to pool quantifiable data from the previous MAs.

**Methods:**

This umbrella review searched the PubMed, Embase and Web of Science databases through June 2026 in accordance with PRISMA recommendations. The MAs that looked at the relationships between third molars and mandibular fractures were included. The odds ratios (OR) and risk ratios (RR) for angle and condylar fractures were the main extraction data. The Newcastle-Ottawa Scale (NOS) criteria were applied for quality assessment and the GRADE tool was utilized to evaluate evidence certainty.

**Results:**

A total of 8 MAs with ∼40 thousand participants were included. Due to the high overlap across these MAs, only 32 unique primary studies were selected from these reviews. Further, an additional 10 studies were included from an independent literature search. In total, 42 studies encompassing ∼16 thousand participants were included for an updated meta-analysis. Third molars were associated with a significantly higher observed risk of mandibular angle fractures (RR 2.18, 95% CI 1.66–2.87, *p* < 0.0001) but they were associated with a significantly lower observed risk of condylar fractures (OR 0.28, 95% CI 0.19–0.43, *p* = 0.001). Most of the subgroup and sensitivity analyses showed the consistency of the results. However, both outcomes received “very low” rate of evidence certainty, mostly due to the observational nature of the included studies, significant heterogeneity and publication bias.

**Conclusions:**

Third molars are associated with a higher observed risk of mandibular angle fractures, but they were associated with a lower observed risk of condylar fractures. Clinical decision on the prophylactic removal of the third molar should balance the risks of angle vs. condylar fractures. Definitive preventive strategies require well-designed prospective research.

**Systematic Review Registration:**

The protocol of this review is registered in PROSPERO database with the registration number of CRD420251121903.

## 1 Introduction

Mandibular fractures are among the most frequent facial injuries, making up roughly 35%–70% of all maxillofacial fractures (1–3). The frequency of mandibular fractures varies from 12.6 to 41.5 incidents per 100,000 people per year (1). At a male-to-female ratio of roughly 3–4:1, these injuries primarily impact young males, with a peak incidence in the second and third decades of life (4). Of all mandibular fractures, 20%–30% are angle fractures, while 30%–40% are condylar fractures (5–7). Fractures frequently develop in the condylar areas and mandibular angle. With different proportional frequency by geographic region and socioeconomic conditions, the main etiological factors are falls, car crashes, interpersonal violence, and injuries sustained in sports (4).

By altering the mandibular structure biomechanically, the presence of third molars has long been thought to affect the risk of mandibular fractures. Third molars take up a large amount of osseous space in the mandibular angle region, which could result in structurally weak spots that are more likely to fracture under traumatic stresses (8). On the other hand, the intricate biomechanics of the mandible imply that third molars may also redistribute stress patterns in ways that could prevent fractures in certain anatomical areas (9). Considering that third molar impaction affects 16%–68% of the population (10, 11), with the highest prevalence recorded in industrialized nations, it is imperative to comprehend this link for both fracture prevention methods and the best possible treatment planning.

Previous systematic reviews (SRs) and meta-analyses (MAs) have looked at different elements of this association, including the impact of third molar presence/absence. However, the evidence has remained dispersed over several SRs with different approaches to methodology and occasionally contradicting findings. Therefore, this umbrella review with updated meta-analysis has two aims: first, to identify and appraise existing systematic reviews and meta-analyses on this association; and second, to conduct an updated meta-analysis of unique primary studies extracted from those reviews, supplemented by an independent search for newer studies, in order to provide a current evidence base to guide clinical decisions on prophylactic third molar removal or retention in patients at risk of mandibular fracture.

## 2 Material and methods

### 2.1 Protocol

The 2020 PRISMA standards were followed in conducting this umbrella review (12), and relevant methodological recommendations for umbrella reviews (13, 14). The protocol for this study was prospectively registered under the registration number of (CRD420251121903) in the PROSPERO database.

### 2.2 Inclusion and exclusion criteria

The PECOS/T framework was used to formulate the research question of this umbrella review. The population was defined as patients with mandibular fractures. The exposure was the presence of mandibular third molars. The comparison was patients with mandibular fractures without third molars. The outcomes of interest were the risk of mandibular angle fracture, the risk of mandibular condylar fracture. Only SRs and MAs reporting results as OR or RR with 95% confidence intervals and published in English up to June 2026 were considered.

All MAs that reported the post-fracture complication and management of third molars in fracture line were excluded. Similarly, those papers which studied one variable (exposure or outcome) without the other, were omitted.

### 2.3 Literature search

The electronic databases of PubMed/MEDLINE, Embase and Web of Science were searched to discover pertinent MA and SRs assessing the research issue**.** All records published prior to June 2026 were included in the search.

Third molar, mandibular fractures (and their subtypes), and systematic review/meta-analysis keywords were combined with controlled vocabulary (MeSH terms) to create a search strategy. Supplementary Table 1 contains the complete list of search phrases and combinations. Only English articles that satisfied the inclusion requirements were included, however there were no limitations on publication dates.

### 2.4 Selection of studies

After entering all of the records that were retrieved from databases into reference management software (Zotero), duplicate records were removed. The two steps of the study selection procedure were independently completed by two reviewers, M. M. and S. O. Using eligibility criteria, abstracts and titles of potentially pertinent studies were screened in the first stage of the two-part study selection process. In Part 2, full-text papers of possibly eligible studies underwent a thorough eligibility check. MAs and SRs who met all eligibility criteria were selected for inclusion. Any differences that came up throughout the screening process were discussed and resolved by the two reviewers. When no agreement could be reached, the ultimate judgement was made by a third reviewer (M. A.). Further, the reference list of the selected studies was searched manually for additional literature, but the grey literature was not searched. There was no need to contact authors as there was no missing or not clear data.

Concerning the updated meta-analysis, all the unique studies in the included MAs were assessed for initial inclusion and full-text appraisal. However, the inclusion criteria were strictly applied to only include those studies that fulfilled the PICOT elements of the research question. Further, since the last included MA was published in 2018, a new literature search was conducted in the same mentioned databases, using the same keywords and applying the same inclusion criteria.

### 2.5 Extraction of data

A structured Microsoft Excel spreadsheet made specifically for this review was used to extract data. Two reviewers (M. M. and S. O.) separately extracted the data in order to minimize variability and the possibility of errors. When the agreement could not be reached, a third reviewer (M. A.) was consulted in order to reconcile the conflicting data extraction.

Sample size, number of third molars (with the total number of fractures), year of publication, number of original records included in the quantitative/qualitative analysis, study design type of the original studies, first author's surname, and key findings were extracted from the studies. Since the main effect size of this review was OR and RR, these were extracted for the pooled analysis.

### 2.6 Synthesis and analysis

The DerSimonian and Laird random-effects model was used for the pooled analyses (15). Further, The Knapp-Hartung adjustment was applied. Since all the outcomes were in OR and RR, these measures were taken and reanalyzed.

The I^2^ statistical test was used to evaluate study heterogeneity. For heterogeneity, a statistically significant *p*-value was defined as less than 0.05. The Cochrane Handbook for Systematic Reviews of Interventions’ recommendations for interpreting I^2^ values were as follows: values between 0% and 40% were deemed possibly inconsequential, moderate heterogeneity was indicated by 30% to 60%, substantial heterogeneity by 50% to 90%, and substantial heterogeneity by 75% to 100% (16).

In addition, a statistical analysis was conducted to determine whether publication bias was present. To detect small-study effects, Egger's regression test was used. A *p*-value of less than 0.05 indicated bias (17). However, since most of the performed pooled analyses contained few studies (<10), we couldn't assess the publication bias for all the outcomes. All statistical analyses in this umbrella review were done online using Cochrane's RevMan program (18).

### 2.7 Evidence certainty

The degree of certainty in the evidence for the outcomes were evaluated using the GRADE approach (19).

### 2.8 Overlap analysis

A Corrected Covered Area (CCA) analysis was conducted to assess the number of repeated primary studies included in the MAs (20).

### 2.9 Study quality assessment

Due to the high overlap rate across the included MAs, only the unique primary studies were selected for the quantitative analysis. The methodological quality of the primary studies included across the eight MAs was assessed using the Newcastle–Ottawa Scale (NOS) for observational studies. First, two authors independently rated the quality, then an average is taken. In case of disagreement, the third author and the quality assessment tables of the included MAs were consulted until the dispute is settled. NOS classifies studies as high quality (≥7 stars), moderate quality (6 stars), or low quality (≤5 stars). The score for each sub-item was averaged across assessments and rounded down at 0.5, consistent with the NOS principle that a star must be clearly demonstrated rather than assumed. Sub-item scores were mapped to three unified domains: Selection (maximum 4 stars), Comparability (maximum 2 stars), and Exposure/Outcome (maximum 3 stars). One study (21) was published only as a conference abstract; no full text was available, and its overall quality rating was therefore derived from the included MA (22).

### 2.10 Meta-regression analysis

To explore potential sources of heterogeneity, meta-regression analyses were performed on the primary study data using a random-effects weighted least squares approach with DerSimonian–Laird tau^2^ estimation. Separate meta-regressions were conducted for the angle and condyle fracture outcomes. For each outcome, the following study-level moderators were tested individually in univariable models: publication year (continuous), sample size (log-transformed, continuous), methodological quality (continuous), and fracture mechanism (categorical) for the angle fracture outcome only.

## 3 Results

### 3.1 Selection of the included reviews

**Figure 1:**
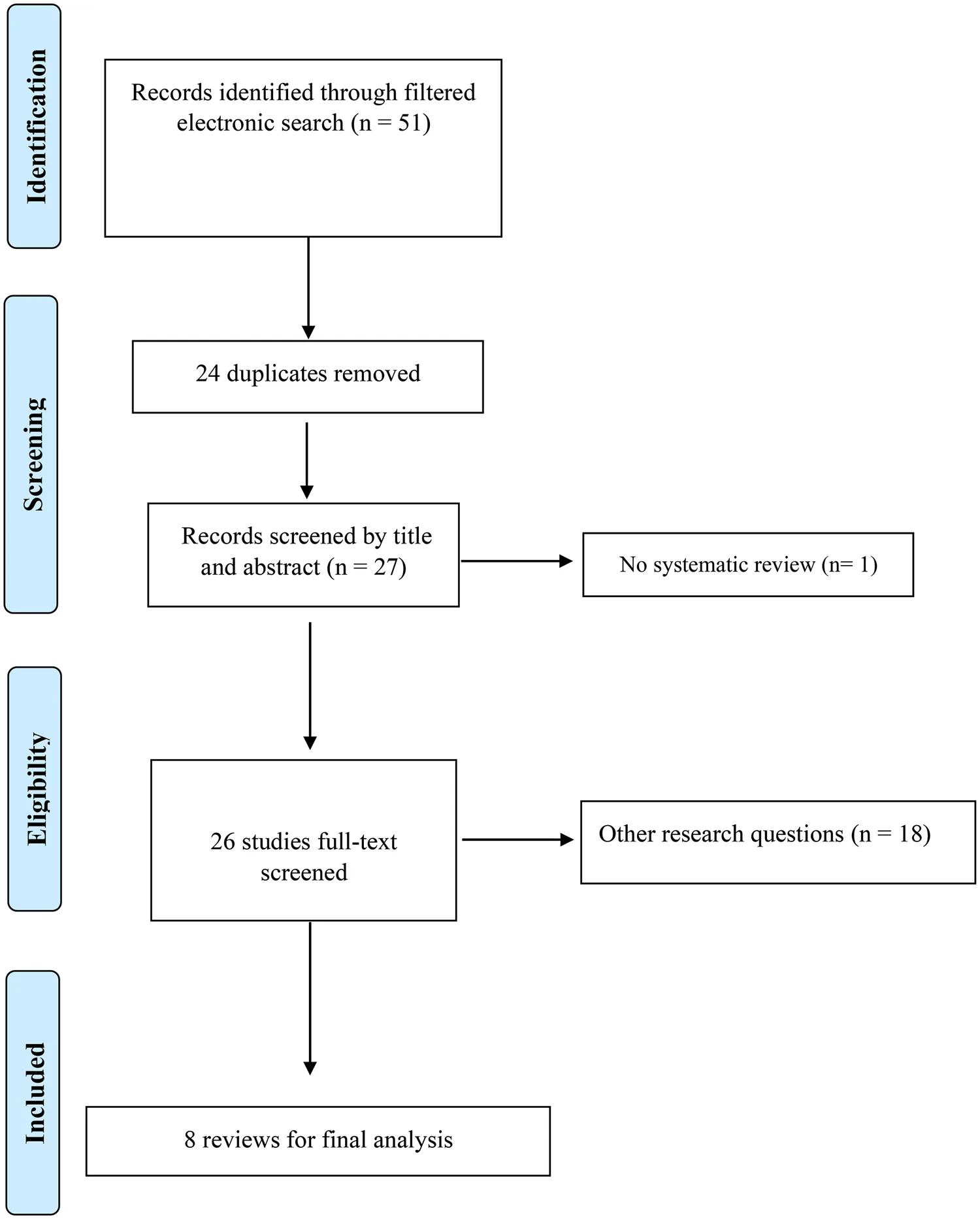
Study selection process of the umbrella review.

A systematic and filtered search of PubMed, Embase and Web of Science yielded 51 records. After removing 24 duplicates, 27 records were screened by title and abstract; one was excluded for not being a systematic review, leaving 26 studies for full-text screening. After applying the inclusion/exclusion criteria, 8 reviews qualified for the final analysis (5–7, 22–25). Supplementary Table 2 shows the list of the included and excluded studies with the reasons of exclusion. Figure 1 shows study selection.

### 3.2 Characteristics of the included reviews

Eight MAs investigated the relationship between third molars and mandibular fractures, with approximately 40 thousand participants. The number of participants in the included MAs ranged from 823 to 13,450, and the follow-up times and methodological strategies varied from one study to the next. Two MAs focused on condylar fractures and found that third molars had a protective impact (OR 0.26–0.35), while six MAs concentrated on mandibular angle fractures and consistently showed an elevated fracture risk with third molar presence (OR/RR ranging from 1.44 to 3.83). Third molar eruption status and location had a major impact on fracture patterns; Class A and Class I positions seemed to be protective, while Class B, Class II, and Class III positions were frequently linked to a higher angle fracture risk. Table 1 shows the main features of these studies.

**Table 1:** Key characteristics of the included reviews.

Studies	No. of included studies in the MA	Total no. of patients	No. of fracture cases with third molar	No. of fracture cases without third molar	Key findings
Armond (2017) (6)	28	13,450 M: 10,960 F: 2,404	4,124	918	Angle fracture: The likelihood of an angle fracture is increased when a mandibular third molar is present: OR = 3.83, (3.02–4.85). Class B (OR 1.44, 95% CI 1.06–1.96) and class II (OR 1.67, 95% CI 1.36–2.04) had the best third molar locations for angle fracture. Angle fracture was prevented by class A (OR 0.60, 95% CI 0.45–0.81) and class I (OR 0.51, 95% CI 0.37–0.71).
Armond (2017) (7)	13	3,814 M: 3,010 F: 718	745	927	Condylar fracture: OR 0.26 (95% CI 0.17–0.40, Classes with the highest risks for condylar fracture: Class A: OR 1.32 (95% CI 1.09–1.61), Class I: OR 1.37 (95% CI 1.05–1.77). Condylar fracture prevention factors: Class B: OR 0.69 (95% CI 0.49–0.97) Class II: OR 0.71 (95% CI 0.57–0.87).
Bezerra (2011) (26)	4	2,533 M: 2,007 F: 526	528	104	Angle fracture: The risk of mandibular angle fracture was 1.94 times higher in those with a third molar (95% CI 1.60–2.35).
Giovacchini (2018) (23)	7	6,750 M: 5,231 F: 1,517	1,852	477	Angle fracture: RR: 1.90 (95% CI = 1.47–2.46) for mandibular angle fracture with third molar presence. Significantly increased risk with Class C (OR 2.72, 95% CI 1.78–4.16), Class II (OR 2.21, 95% CI 1.69–2.87), and Class III (OR 2.99, 95% CI 2.12–4.22) third molar positions.
Hanson (2004) (22)	6	3,002 M: 2,212 F: 575	686	149	Angle fracture: RR = 2.8 (95% CI 2.3–3.5)
Machete (2016) (24)	19	9,888	NS	NS	Angle fracture: RR: 1.44 (95% CI 1.31–1.57) for mandibular angle fracture with third molar presence, representing a 44% increase in risk.
Ruela (2017) (25)	16	7,720	1,862	506	Angle fracture: Mandibular angle fractures were 3.16 times more common in lower third molars (OR 3.16, 95% CI 2.20–4.54). Highest risk positions: Class III horizontal (59.84%) and Class C vertical (63.67%)
Xu (2017) (5)	13	2,399	1,893	506	Angle fracture: The incidence of mandibular angle fractures was 2.63 times higher in patients with third molars (RR 2.63, 95% CI 2.15–3.21).
Xu (2017) (5)	5	823	355	468	Condylar fracture: Patients with third molars have 0.47 times lower risk of condylar fractures (RR 0.47, 95% CI 0.25–0.86).

### 3.3 Overlap analysis results

The corrected covered area (CCA) was calculated to quantify the degree of primary study overlap across the included MAs. For the mandibular angle fracture outcome, CCA indicated very high overlap of 53%. For the mandibular condylar fracture outcome, overlap between the two contributing MAs was high (13%). Across all eight estimates combined, overall overlap was very high (60%) (Supplementary Table 3). Due to this high overlap, we decided to conduct an updated meta-analysis including the unique studies only.

### 3.4 Updated meta-analysis

#### 3.4.1 Eligibility criteria

Eligible studies were case-control or cross-sectional studies reporting the association between mandibular third molar presence and mandibular angle or condyle fractures, with a valid comparator group and sufficient data at the patient level. Studies comparing eruption states only without an absent third molar control arm, or lacking raw cell counts from any accessible source, were excluded.

#### 3.4.2 Study selection

A total of 120 primary studies were used across the 8 included MAs. However, only 41 studies were unique. Supplementary Table 4 shows the matrix overlap of the primary studies.

**Figure 2:**
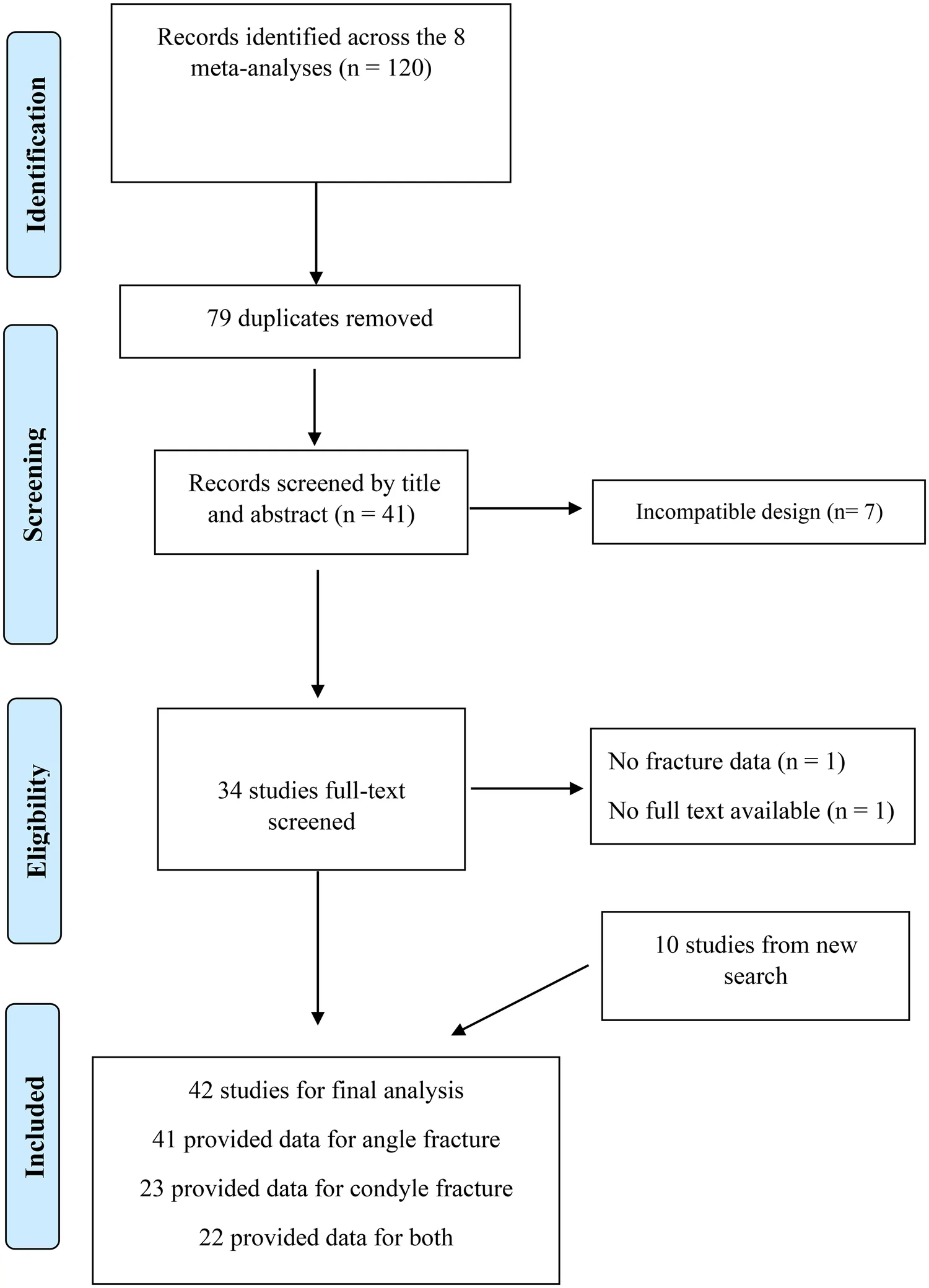
Study selection process for the updated meta-analysis.

Among the 41 unique studies, 7 were excluded due to incompatible design, one for lack of fracture data and one due to full-text non-availability. Supplementary Table 5 shows the excluded studies and their reasons. Thirty-two studies were qualified to be included in the updated meta-analysis (8, 21, 27–56). Moreover, a literature search was conducted independent of the included MAs and this revealed another 10 primary studies published after the last MA on the topic (57–66). Finally, 42 unique studies were included. Among these, 41 and 23 of them provided data for angle and condyle fracture meta-analyses, respectively. Twenty-two studies provided data for both. Table 2 shows the characteristics of the included primary studies. Figure 2 shows the study selection process for the updated meta-analysis.

**Table 2:** Characteristics of the included primary studies.

Author	Year	Country	N	NOS Quality	OR (95% CI)	RR (95% CI)
**Mandibular Angle Fracture Studies (*n*** **=** **41)**						
Tankersly and Abubaker (21)	1995	USA	215	Low	5.73 (3.10–10.60)	—
Tevepaugh and Dodson (27)	1995	USA	101	Moderate	—	3.88 (1.30–11.60)
Safdar and Meechan (28)	1995	UK	200	Moderate	—	2.17 (1.59–2.95)
Yamada et al. (29)	1998	Japan	30	Low	26.87 (1.52–475.11)	—
Lee and Dodson (30)	2000	USA	367	Moderate	—	1.87 (1.20–2.90)
Ugboko et al. (31)	2000	Nigeria	490	Moderate	—	1.18 (0.65–2.14)
Ma’aita and Alwrikat (32)	2000	Jordan	615	Moderate	—	2.25 (1.52–3.33)
Fuselier et al. (33)	2002	USA	1,210	Moderate	—	2.10 (1.62–2.72)
Kasamatsu et al. (34)	2003	Japan	151	Moderate	—	3.42 (1.70–6.90)
Kim (35)	2004	South Korea	107	Moderate	6.90 (2.38–19.99)	—
Halmos et al. (36)	2004	USA	1,450	High	—	2.80 (2.30–3.40)
Iida et al. (37)	2004	Japan	346	Moderate	4.48 (2.74–7.33)	—
Iida et al. (38)	2005	Germany	218	Moderate	—	2.16 (1.23–3.78)
Zhu et al. (39)	2005	South Korea	439	Low	5.42 (3.56–8.24)	—
Cho et al. (40)	2006	South Korea	546	Moderate	3.30 (2.18–5.00)	—
Duan and Zhang (42)	2008	China	700	High	—	1.78 (1.57–2.02)
Park et al. (55)	2009	South Korea	205	Moderate	2.90 (1.63–5.15)	—
Rajkumar et al. (43)	2009	India	154	Low	—	1.11 (1.00–1.23)
Subhashraj (44)	2009	India	2,033	High	—	1.10 (0.93–1.31)
Thangavelu et al. (45)	2010	India	460	Moderate	—	2.31 (1.78–3.00)
Choi et al. (46)	2011	South Korea	385	High	6.74 (4.19–10.83)	—
Lee et al. (47)	2012	South Korea	86	Moderate	4.63 (1.97–10.90)	—
Patil (48)	2012	India	190	High	—	1.19 (1.08–1.31)
Rajandram et al. (50)	2013	Malaysia	214	High	—	3.57 (2.49–5.12)
Gaddipati et al. (51)	2014	India	110	High	—	37.27 (12.77–108.78)
Naghipur et al. (52)	2014	Canada	446	High	—	1.88 (1.51–2.33)
Mah et al. (53)	2015	South Korea	440	High	—	6.58 (3.51–12.33)
Rahimi-Nedjat et al. (54)	2016	Germany	1,219	Moderate	3.77 (2.63–5.40)	—
Nogami et al. (59)	2018	Japan	241	Moderate	7.25 (4.06–12.95)	—
Mehra et al. (63)	2019	India	280	Moderate	51.37 (17.93–147.21)	—
Lee Y et al. (65)	2019	South Korea	129	High	2.24 (1.07–4.72)	—
Soós et al. (67)	2020	Hungary	279	High	2.18 (1.32–3.61)	—
Brucoli et al. (58)	2020	Italy	118	Moderate	8.28 (3.26–21.03)	—
Al-Sharani et al. (66)	2021	China/Yemen	73	Low	2.94 (1.11–7.80)	—
Venkatachalam et al. (61)	2022	India	85	Low	2.08 (0.86–5.06)	—
Mu et al. (62)	2024	China	184	Moderate	3.03 (1.49–6.18)	—
Rozenbilds et al. (62)	2026	Australia	475	Moderate	3.32 (2.23–4.94)	—
Al-Harbawee et al. (68)	2021	UK	482	High	4.26 (2.59–7.00)	-
Meisami et al. (56)	2002	Canada	413	Moderate		2.80 (1.49–5.27)
Antic et al. (8)	2016	Serbia	615	Moderate	—	1.88 (1.45–2.44)
Luria and Campbell (49)	2013	USA	234	Low	—	2.09 (1.31–3.35)
**Mandibular Condylar Fracture Studies (*n*** **=** **23)**						
Iida et al. (37)	2004	Japan	346	Moderate	0.45 (0.31–0.64)	—
Zhu et al. (39)	2005	South Korea	439	Low	0.23 (0.15–0.34)	—
Oh et al. (41)	2006	South Korea	105	Low	0.58 (0.35–0.97)	—
Duan and Zhang (42)	2008	China	700	High	0.54 (0.40–0.74)	—
Thangavelu et al. (45)	2010	India	460	Moderate	—	0.44 (0.34–0.56)
Choi et al. (46)	2011	South Korea	385	High	0.29 (0.19–0.45)	—
Lee et al. (47)	2012	South Korea	86	Moderate	0.25 (0.09–0.72)	—
Patil (48)	2012	India	190	High	0.12 (0.03–0.51)	—
Luria and Campbell (49)	2013	USA	234	Low	—	2.08 (1.30–3.32)
Gaddipati et al. (51)	2014	India	110	High	0.03 (0.01–0.08)	—
Naghipur et al. (52)	2014	Canada	446	High	—	0.57 (0.41–0.79)
Mah et al. (53)	2015	South Korea	440	High	0.30 (0.18–0.50)	—
Antic et al. (8)	2016	Serbia	615	Moderate	0.70 (0.50–0.97)	—
Nogami et al. (59)	2018	Japan	241	Moderate	0.14 (0.08–0.25)	—
Mehra et al. (63)	2019	India	280	Moderate	0.02 (0.01–0.06)	—
Lee Y et al. (65)	2019	South Korea	129	High	0.45 (0.21–0.94)	—
Soós et al. (67)	2020	Hungary	279	High	0.46 (0.28–0.76)	—
Brucoli et al. (58)	2020	Italy	118	Moderate	0.12 (0.05–0.31)	—
Al-Sharani et al. (64)	2021	China/Yemen	73	Low	0.34 (0.13–0.91)	—
Venkatachalam et al. (66)	2022	India	85	Low	0.48 (0.20–1.17)	—
Mu et al. (61)	2024	China	184	Moderate	0.33 (0.16–0.67)	—
Rozenbilds et al. (62)	2026	Australia	475	Moderate	0.69 (0.46–1.04)	—
Al-Harbawee et al. (57)	2021	UK	482	High	0.24 (0.14–0.39)	—

OR, odds ratio; RR, risk ratio; CI, confidence interval; NOS, Newcastle–Ottawa Scale. Twenty-two studies appear in both sections, giving 42 unique primary studies across both outcomes.

#### 3.4.3 Data extraction

Data were collected directly from the tables and forest plots of the included MAs. In few cases, raw cell counts (a, b, c, d) were extracted directly from the original primary studies where accessible. The OR was calculated as (a × d)/(b × c) and the risk ratio (RR) as [a/(a + c)]/[b/(b + d)], with 0.5 continuity correction applied to zero cells. Ninety-five percent confidence intervals were derived using the Woolf method for OR and the log-binomial method for RR.

#### 3.4.4 Quality assessment results

Quality was assessed by NOS. The total scores across the 42 primary studies ranged from 5 to 8. Thirteen studies were rated high quality (≥7 stars), twenty-one were rated moderate quality (6 stars), and eight were rated low quality (≤5 stars). Across all domains, the Exposure/Outcome domain was consistently awarded full marks (3/3 stars) in all studies, reflecting uniform use of validated radiographic ascertainment and appropriate statistical methods. The Comparability domain showed the greatest variability and was the most commonly deficient, with the majority of studies failing to control for confounding variables. Within the Selection domain, the definition of controls (case–control studies) and outcome not present at start of study (cohort studies) were the sub-items most frequently problematic. Full details of the quality evaluation are presented in Supplementary Table 6.

### 3.5 Updated meta-analysis results

Overall, we conducted a total of four main, pooled analyses and several subgroup and sensitivity analyses. Details of the findings are presented in Table 3.

**Table 3:** Meta-analysis results of the association of mandibular angle and condyle fractures with the presence of third molars.

Variables	No. of included estimates	Effect size (95% CI)	*p*-value	I^2^ (95% CI), p heterogenicity	p Egger's test (Publication bias)	GRADE
Angle fracture						
RR	21	2.18 (1.66, 2.87)	*p* < 0.0001	91.7%, *p* < 0.0001	0	Very low
Trim and fill (RR)	31	1.45 (1.03, 2.04)	0.03	93.4%, *p* < 0.0001		
OR	20	4.40 (3.29, 5.88)	*p* < 0.0001	64.5%, *p* < 0.0001	0.18	Very low
*Sample size (RR)*	21		Intergroup *p* = 0.46			
Large (*N* > 300)	11	2.01 (1.53, 2.65)				
Small (*N* < 300)	10	2.48 (1.38, 4.46)				
*Study quality (RR)*	21		Intergroup *p* = 0.54			
High quality	9	2.21 (1.16, 4.20)				
Low quality	2	1.57 (0.02, 161.14)				
Moderate quality	10	2.36 (1.92, 2.90)				
*Time (RR)*	21		Intergroup *p* = 0.02			
<2000	5	2.03 (1.41, 2.93)				
2001–2005	5	2.54 (2.06, 3.12)				
2006–2010	4	1.48 (0.83, 2.63)				
2011–2016	7	2.98 (1.17, 7.58)				
*Fracture cause (RR)*	21		Intergroup *p* = 0.72			
Not reported	3	2.40 (1.55, 3.72)				
RTA	10	1.94 (1.12, 3.39)				
Violence	8	2.34 (1.69, 3.24)				
Condyle fracture						
OR	20	0.28 (0.19, 0.43)	*p* < 0.0001	83.9%, *p* < 0.0001	0.003	Very low
Trim and fill (OR)	28	0.47 (0.28, 0.78)	0.005	89.5%, *p* < 0.0001		
RR	4	0.78 (0.27, 2.24)	0.51	92%, *p* < 0.0001		Very low
*Sample size (OR)*	20		Intergroup *p* = 0.83			
Large (*N* > 200)	12	0.29 (0.16, 0.51)				
Small (*N* < 200)	8	0.26 (0.12, 0.59)				
*Study quality (OR)*	9		Intergroup *p* = 0.31			
High quality	8	0.28 (0.13, 0.62)				
Moderate quality	7	0.20 (0.07, 0.58)				
Low quality	5	0.39 (0.24, 0.64)				
*Time (OR)*	20		Intergroup *p* = 0.23			
2001–2010	4	0.42 (0.21, 0.83)				
2011–2017	6	0.22 (0.07, 0.68)				
2018–2026	10	0.25 (0.12, 0.51)				
*Fracture cause (OR)*	20		Intergroup *p* = 0.16			
Not reported	4	0.22 (0.08, 0.62)				
RTA	7	0.18 (0.05, 0.68)				
Violence	9	0.38 (0.27, 0.53)				

CI, confidence interval; OR, odds ratio; RR, risk ratio; RTA, road traffic accident; NR, not reported.

#### 3.5.1 Angle fracture

**Figure 3:**
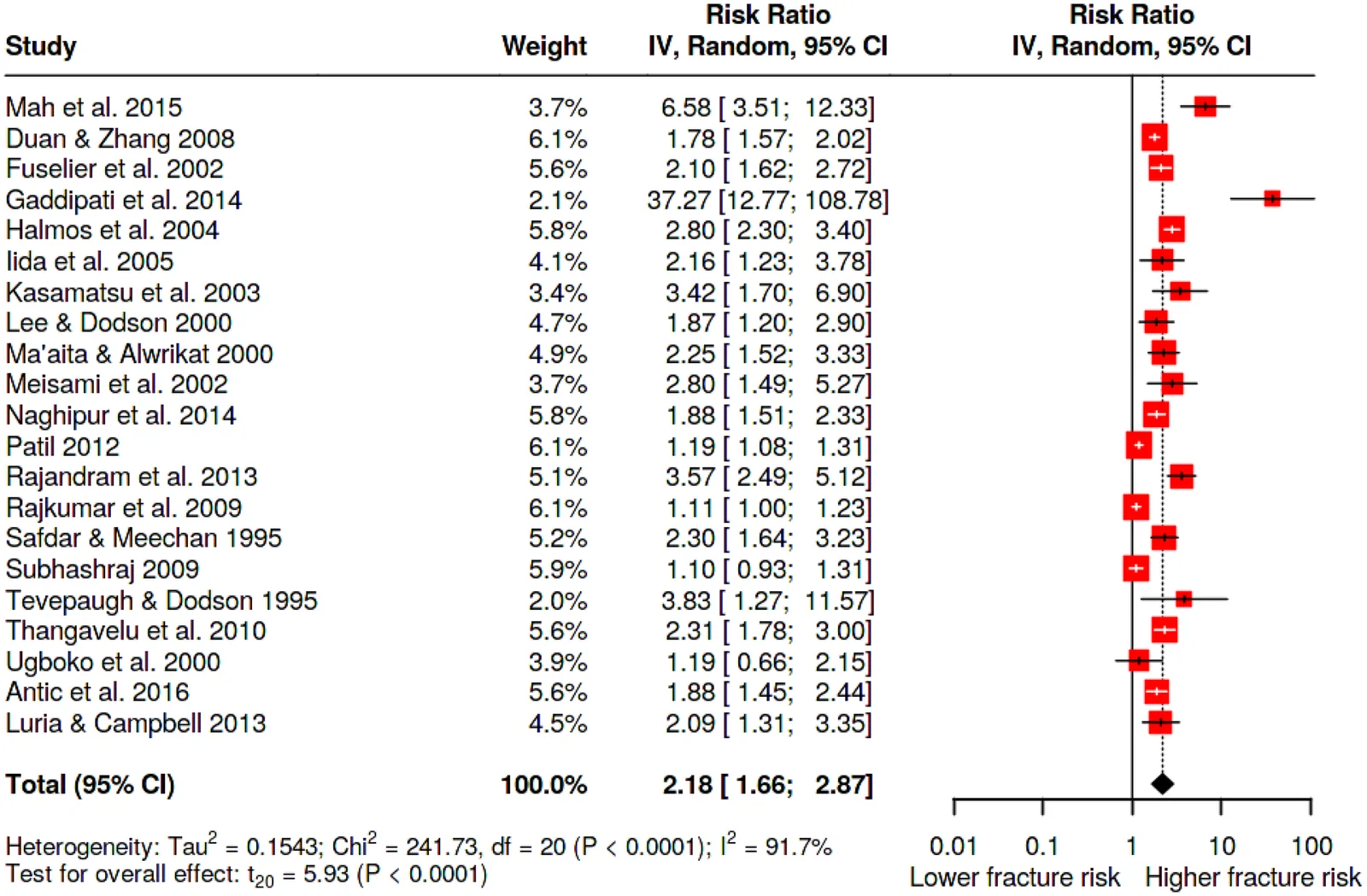
Forest plot of the risk ratio of mandibular angle fractures in the presence of third molars.

**Figure 4:**
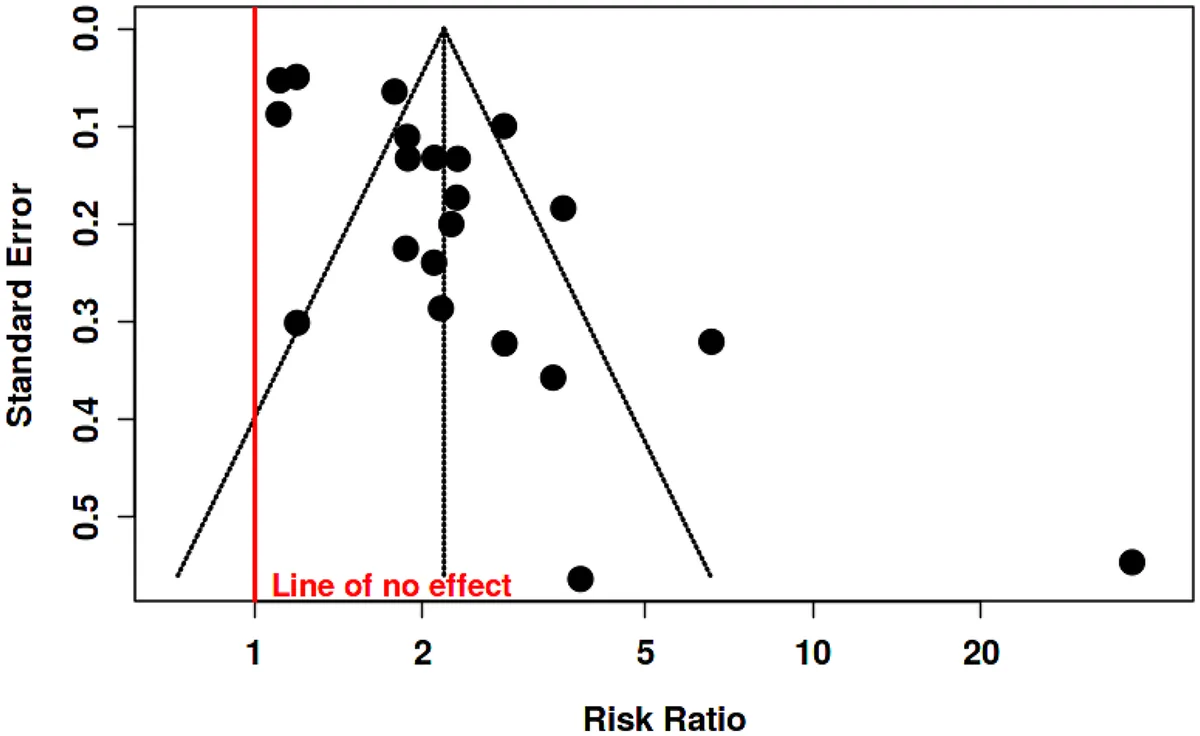
Funnel plot of the risk ratio of mandibular angle fractures in the presence of third molars showing significant publication bias.

**Figure 5:**
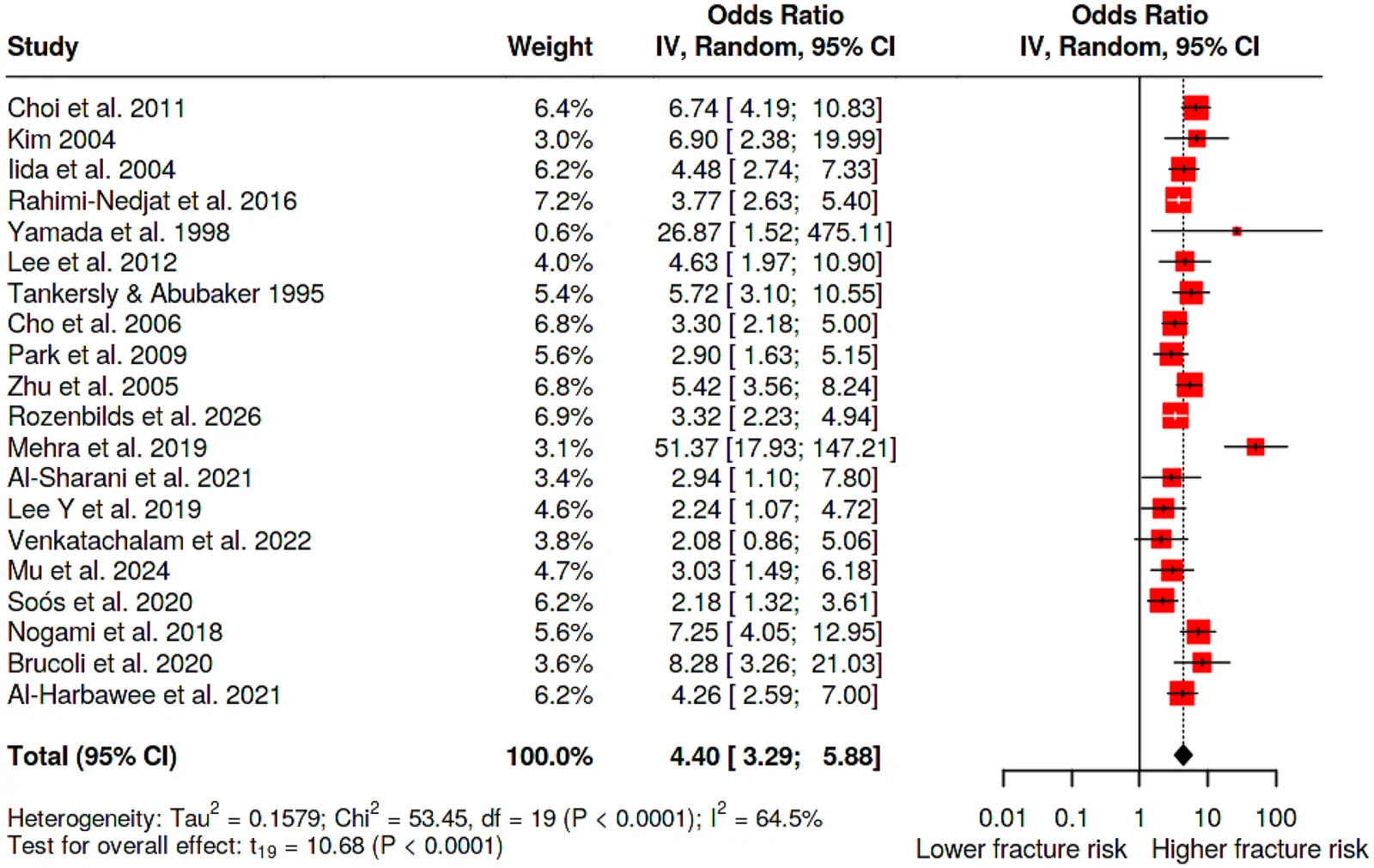
Forest plot of the odds ratio of mandibular angle fractures in the presence of third molars.

**Figure 6:**
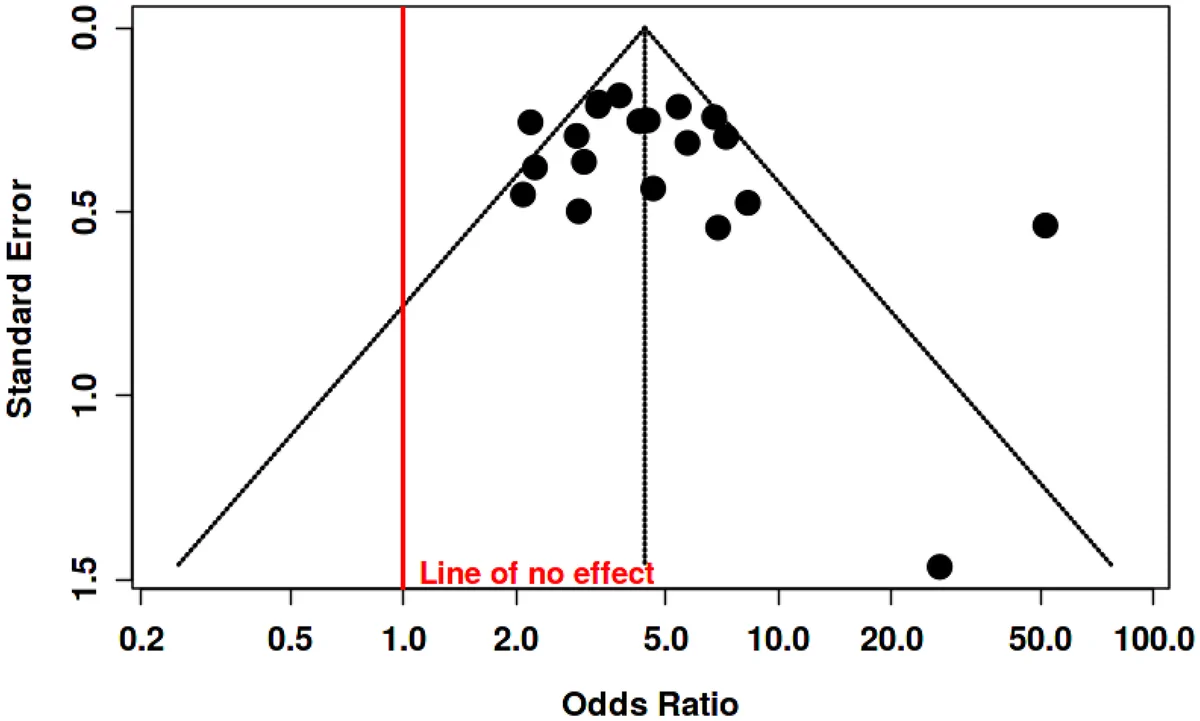
Funnel plot of the odds ratio of mandibular angle fractures in the presence of third molars showing non-significant publication bias.

The third molars were linked to a two-fold higher risk of angle fractures when comparing RR values from 21 estimates (RR 2.18, 95% CI 1,66–2.87, *p* < 0.0001) but with significant heterogeneity and publication bias (Table 3, Figures 3, 4). To compensate the publication bias, a trim and fill analysis was conducted. Although this reduced the effect size but still remained significant (RR 1.45, 95% CI 1,03–2.04, *p* = 0.03) (Table 3, Supplementary Figure 1). The leave-one out sensitivity analyses showed the consistency of the findings (Supplementary Table 7). An even greater correlation was seen when ORs were analyzed (20 estimates: OR 4.40, 95% CI 3.29–5.88, *p* < 0.0001), with substantial heterogeneity but no significant publication bias (Figures 5, 6).

The evidence for angle fracture was graded as very low certainty, downgraded primarily due to serious risk of bias inherent to retrospective observational designs, substantial statistical heterogeneity, and suspected publication bias. Details of the GRADE assessment are presented in the Supplementary Table 8.

The RR measurement was used for the subgroup analyses. Generally, most of the findings showed the consistency of the results. No significant difference was found between large sample size compared with small sample size studies (Table 3, Supplementary Figure 2). For the study quality, contrary to moderate and high-quality studies low-quality studies failed to show a significant result. However, the subgroup difference between the three groups was not significant (*p* = 0.75) (Table 3, Supplementary Figure 3). In the time-wise analysis, all the groups except 2006–2010 group showed statistically significant results (Table 3, Supplementary Figure 4). Lastly, the subgroup analysis based on the fracture cause revealed that the association was highest for violence, followed by road traffic accident and “not reported” category (Table 3, Supplementary Figure 5).

#### 3.5.2 Condylar fracture

**Figure 7:**
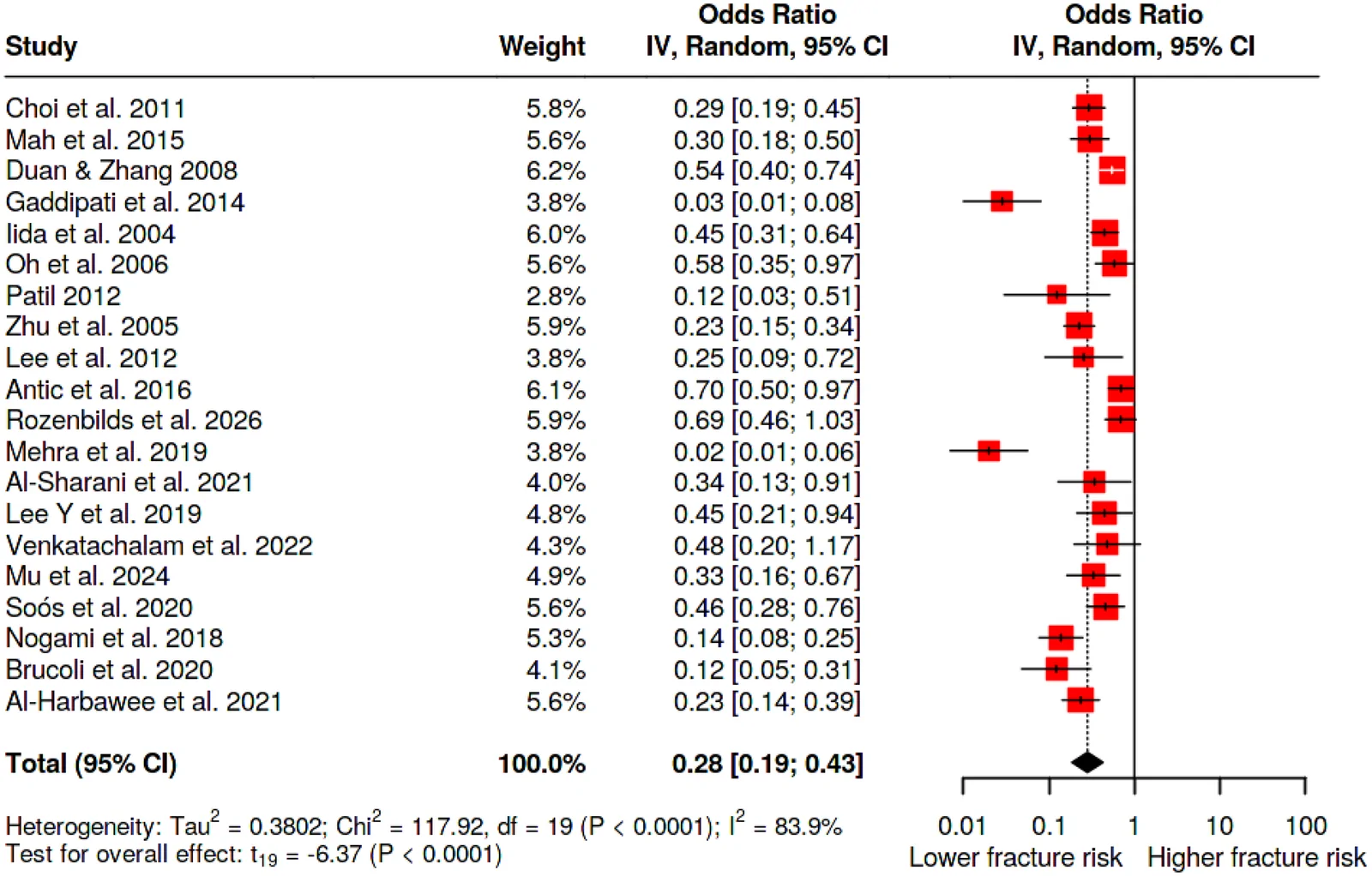
Forest plot of the odds ratio of condylar fracture in the presence of third molars.

**Figure 8:**
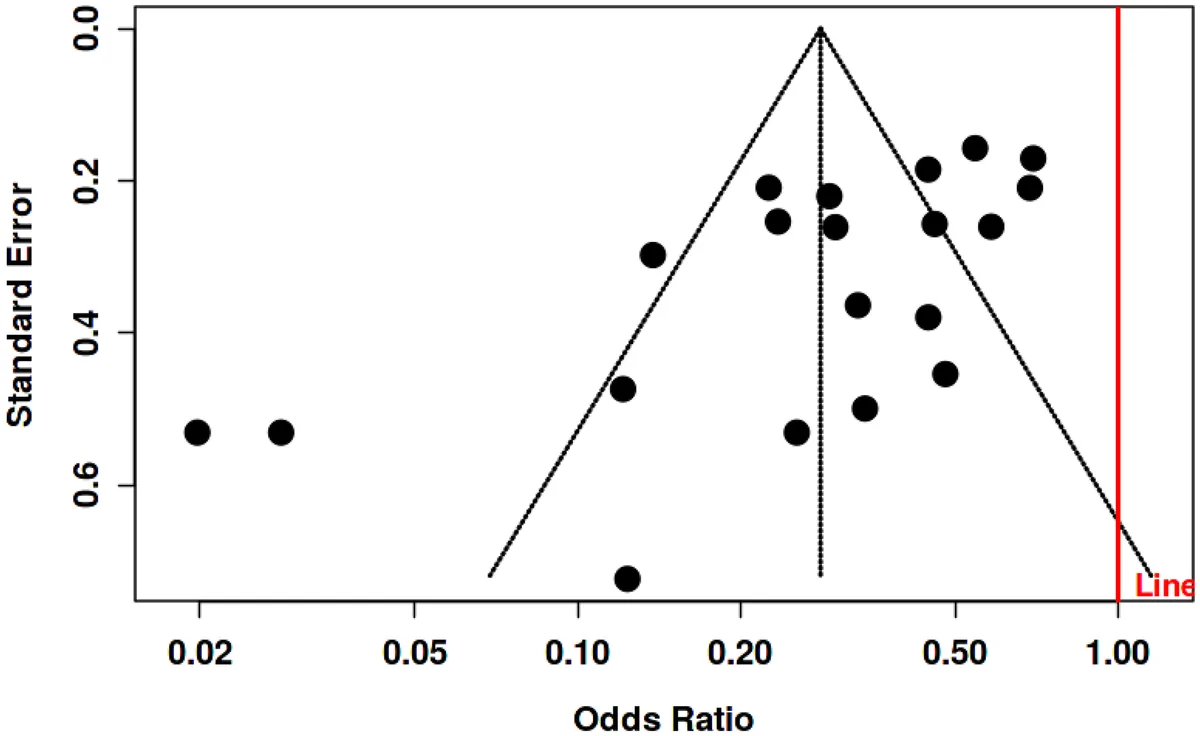
Funnel plot of the odds ratio of condylar fracture in the presence of third molars showing significant publication bias.

The analysis of 20 OR estimates demonstrated that presence of third molars was associated with a significantly lower risk of condylar fractures (OR 0.28, 95% CI 0.19–0.43, *p* < 0.0001), but with significant heterogeneity and publication bias (Table 3, Figures 7, 8). In the trim and fill analysis, the effect size increased (28 estimates: OR 0.47, 95% CI 0.28–0.78) (Table 3, Supplementary Figure 6). The leave-one out sensitivity analyses showed the consistency of the findings (Table 3, Supplementary Table 9). Only 4 estimates contributed to the RR meta-analysis showing non-significant results with significant heterogeneity (RR 0.78, 95% CI 0.27–2.24, *p* = 0.51) (Table 3, Supplementary Figure 7).

Evidence of the condylar fracture was graded as very low certainty, downgraded due to serious risk of bias, substantial heterogeneity, and serious imprecision attributable to the limited number of contributing studies. Details of the GRADE assessment are presented in the Supplementary Table 8.

The OR measurements were selected for the subgroup analyses. Generally, the subgroup findings were consistent across all strata. For the sample size comparison, both large and small sample size studies reflected the protective effect of third molar presence against condylar fracture, with no significant difference between subgroups (Table 3, Supplementary Figure 8). Moreover, findings were consistent across all quality strata, with high-, moderate-, and low-quality studies all demonstrating a significant protective association (Table 3, Supplementary Figure 9). There was no significant difference between the pooled results of studies from different time frames, with consistent protective estimates across all three periods (Table 3, Supplementary Figure 10). Lastly, all fracture cause subgroups showed a protective effect, with violence-related fractures showing the highest and most precise estimate, while road traffic accident and not-reported mechanism studies showed wider confidence intervals, though no significant intergroup difference was detected (Table 3, Supplementary Figure 1^1^).

Among the included studies only nine reported Pell & Gregory classification data. Position C was the only subgroup reaching statistical significance, showing a protective effect against angle fracture (OR = 0.64, 95% CI 0.42–0.98) and a reciprocal increase in condyle fracture risk (OR = 1.57, 95% CI 1.02–2.40). Heterogeneity was substantial across most subgroups (I^2^ = 71.9–90.3%) but absent for Position C (I^2^ = 0%), suggesting consistent behavior of deeply impacted third molars across study populations (Supplementary Figures 12, 13).

#### 3.5.3 Meta regression analysis

Univariable meta-regression analyses identified no statistically significant moderator for either outcome. For angle fractures, publication year, sample size, NOS score, and fracture mechanism were each non-significant (all *p* > 0.05), with residual heterogeneity remaining significant across all models (residual Q *p* < 0.005). For condyle fractures, publication year, sample size, and NOS score were all non-significant (all *p* > 0.05), with residual heterogeneity remaining significant across all models (residual Q *p* ≤ 0.054), reflecting the persistence of substantial unexplained heterogeneity even after accounting for these moderators with 20 contributing estimates. Supplementary Tables 10, 11 present the meta-regression results and summary of the residual heterogeneity.

## 4 Discussion

To the best of our knowledge and literature search, this is the first umbrella review of all MAs on the association between third molars and mandibular fractures.

The pattern of mandibular fractures was greatly influenced by the presence of third molars. According to our updated meta-analysis of 42 unique studies with approximately 16 thousand participants, third molars were associated with a significantly increased incidence of angle fractures (RR 2.18, 95% CI 1.66, 2.87). However, the third molars demonstrated a protective effect against condylar fractures (OR 0.28, 95% CI 0.19, 0.43). Leave-one-out sensitivity analyses for both angle and condylar fractures supported the consistency of the findings. Both the angle and condylar fractures received a “very low” certainty of evidence measured by GRADE tool.

Subgroup analyses of angle fracture data revealed that neither sample size (large vs. small; intergroup *p* = 0.93) nor study quality (high, moderate, or low NOS; intergroup *p* = 0.75) significantly modulated the pooled effect, suggesting that the observed two-fold increase in angle fracture risk is robust across methodological and sample-size strata. However, publication year, was the only significant effect modifier (intergroup *p* = 0.008). Studies published between 2001 and 2005 yielded the highest pooled (RR 2.54), whereas those from 2006 to 2010 produced a comparatively attenuated estimate (RR 1.49), with the 2011–2016 cohort showing a wide confidence interval (RR 2.25, 95% CI 1.15–4.39) likely reflecting heterogeneous study designs accumulated over that period. The fracture etiology subgroup did not significantly differ, although violence-related fractures trended toward a higher point estimate (RR 2.34) compared with RTA-related fractures (RR 1.86), possibly reflecting differences in impact force and direction. For condylar fracture, all the subgroups were significant and none of the subgroup analyses of sample size (*p* = 0.83), study quality (*p* = 0.31), publication year (*p* = 0.23), or fracture etiology (*p* = 0.16) reached statistical significance, implying that the protective association of third molar presence against condylar fracture is consistent irrespective of these moderating variables.

In the meta-regression analyses of the angle fractures, none of the tested moderators (publication year, sample size, NOS quality score, or fracture mechanism) significantly predicted the effect size (all *p* > 0.05). Residual heterogeneity remained statistically significant across all models, indicating that the tested variables do not account for the observed between-study variance. For condyle fractures, none of the three variables tested (publication year, sample size, NOS score) reached statistical significance (all *p* > 0.05), and the previously observed borderline trend for sample size (*p* = 0.081 with k = 10) was no longer apparent with the expanded pool of 20 contributing studies (*p* = 0.218), suggesting it was a spurious finding driven by limited statistical power. Residual heterogeneity in the condyle models remained significant across most models (*p* = 0.037–0.054), confirming that substantial between-study variance persists even after accounting for these moderators. Taken together, these findings suggest that the residual heterogeneity in both outcomes is not attributable to any single identifiable study-level characteristic and likely reflects unmeasured clinical and methodological diversity, including differences in fracture definitions, imaging modalities, third molar classification systems, and patient demographics across the primary studies.

There is strong experimental support for the biomechanical explanation of the results of this review. About 60% of the force needed to fracture mandibles with erupted third molars to fracture a mandible with unerupted third molars (54). The mandibular angle region is weakened because third molars take up osseous space that would otherwise store bone (69, 70). A mechanical explanation for the opposing fracture risks is provided by finite element studies, which show that the presence of a third molar raises stress forces at the angle region while decreases them at the condyle (71, 72). Furthermore, the external oblique ridge, which typically gives the mandibular angle region structural support, may be disrupted by partially impacted third molars (73).

The position of the third molar has an impact on fracture patterns. The finding that Position C third molars are significantly associated with reduced angle fracture risk and increased condyle fracture risk is mechanistically plausible, as deeply impacted teeth may redistribute impact forces away from the angle toward the condylar neck. However, due to the small number of contributing studies, these results should be interpreted as hypothesis-generating only. According to Armond et al., class A and class I provided protection against angular fracture, whereas class B and class II increased the likelihood of it (6). Giovacchini et al's meta-analysis found that none of the other classes significantly decreased the relative risk of mandibular angle fracture associated with third molar position. For Class C, Class II, and Class III, the risk was 2.72, 2.21, and 2.99, respectively (23). This suggests that more posterior locations and deeper impacts result in greater structural weakness (45, 73). Nonetheless, some research suggests that Class IIB slightly impacted molars may be more dangerous than fully impacted teeth (74).

The results of this review may have implications for clinical decision-making. Any decision on prophylactic third molar extraction should contain condylar fracture concerns also. Third molar extraction raises the incidence of condylar fractures, despite some advocates for its preventative removal in high-risk groups, for example contact athletes (75). Because prevalence of mandibular angle fractures are not very common (1%–2%) (76, 77), it is challenging to defend preventive and prophylactic extraction based on fracture prevention alone.

The connection between third molars and fracture patterns is also influenced by the mechanism of the injury. When third molars are present, mandibular angle fractures are more commonly linked to low-intensity impacts like physical assaults and sports injuries (60, 78). Regardless of the state of the third molar, condylar fractures are more likely in high-energy trauma (automobile accidents) than angle fractures (54). Age, occlusal condition, and the shape of the third molar root are other variables that influence fracture risk (79–81). The included MAs did not stratify findings by patient-related factors such as age, sex, or bone density, nor did they differentiate between unilateral and bilateral fractures, which may represent biomechanically distinct injury patterns associated with different energy levels of trauma. These represent important gaps in the current literature that future primary studies should address to allow more precise risk stratification.

The overlap analysis revealed a very high level of overlap among the included MAs (53% for angle fracture, 13% for condylar fracture, 60% overall). This means that the same pool of original studies has been reused across the MAs. This indicates that the field would benefit from new original studies with true cohorts rather than reanalyzing the same set of primary studies in repeated MAs. Hence, while this umbrella review consolidates fragmented evidence, the very low certainty of the evidence, substantial heterogeneity, and observed publication bias mean these pooled estimates should be regarded as hypothesis-generating rather than conclusive, and do not by themselves constitute sufficient evidence to support definitive clinical guidance on third molar retention or removal for fracture prevention. Instead, more attention should be paid to the post-fracture complications and clinical guidelines to help in the decision of retention or extraction of third molars in the line of fracture.

### 4.1 Limitations

Most of the included MAs and primary studies covered studies that were retrospective and case controls, and the quality of the evidence differed throughout them. Some studies claimed to be retrospective cohorts, but a true cohort study does not begin with the outcome (mandibular fracture), rather followed patients with specific molar characteristics and compared the outcome in different groups. There was significant heterogeneity, which probably reflected variations in the study populations, trauma causes, and methodology. Moreover, publication bias was significant for some of the pooled analyses. In addition, the scarcity of prospective randomized controlled trials and varied follow-up lengths were other drawbacks. The certainty of the evidence was rated as very low. Finally, the quality of the included studies was mostly moderate, and this might have affected some of the outcomes.

## 5 Conclusions

Within the limitations of this study, it appears that third molars considerably change mandibular fracture patterns. However, the significant heterogeneity across the included studies and the very low evidence certainty prevents any strong clinical conclusions. Given that condylar fractures are more difficult to treat and are linked to higher morbidity, clinical judgments on the prophylactic removal of the third molar should balance the risks of angle vs. condylar fractures. Definitive preventive strategies require well-designed prospective research.

## Data Availability

The original contributions presented in the study are included in the article/Supplementary Material, further inquiries can be directed to the corresponding author.
