# Supplementary material for: Role of third molars in mandibular fractures: a systematic umbrella review with updated meta-analysis
Source: Front Oral Health. 2026 Aug 17;7:1865229. doi: 10.3389/froh.2026.1865229 (PMC13530431; doi:10.3389/froh.2026.1865229)
Supplement: Supplementary file 1 [file Supplementaryfile1.docx]

**Supplementary material for the article:**

**Role of third molars in the mandibular fracture: a systematic umbrella review with updated meta-analysis**

**Supplementary Table 1. Search strategy**

| **Databases** | **Search strategy** |
| --- | --- |
| **PubMed** | (("mandibular fracture*"[Title/Abstract] OR "mandible fracture*"[Title/Abstract] OR "jaw fracture*"[Title/Abstract] OR "mandibular trauma"[Title/Abstract] OR "angle fracture*"[Title/Abstract] OR "condylar fracture*"[Title/Abstract]) AND ("third molar*"[Title/Abstract] OR "wisdom tooth"[Title/Abstract] OR "wisdom teeth"[Title/Abstract] OR "mandibular third molar*"[Title/Abstract] OR "lower third molar*"[Title/Abstract] OR "impacted tooth"[Title/Abstract] OR "impacted teeth"[Title/Abstract] OR "tooth impaction"[Title/Abstract]) AND ("systematic review"[Title/Abstract] OR "meta-analysis"[Title/Abstract] OR "meta analysis"[Title/Abstract] OR "pooled analysis"[Title/Abstract] OR "systematic literature review"[Title/Abstract])) AND English[Language] |
| **Web of science** | (("mandibular fracture*" OR "mandible fracture*" OR "jaw fracture*" OR "mandibular trauma" OR "angle fracture*" OR "condylar fracture*") AND ("third molar*" OR "wisdom tooth" OR "wisdom teeth" OR "mandibular third molar*" OR "lower third molar*" OR "impacted tooth" OR "impacted teeth" OR "tooth impaction") AND ("systematic review" OR "meta-analysis" OR "meta analysis" OR "pooled analysis" OR "systematic literature review")) Quick Filters: Review Article |
| **Embase** | (('mandibular fracture':ti,ab,kw OR 'mandible fracture':ti,ab,kw OR 'jaw fracture':ti,ab,kw OR 'mandibular trauma':ti,ab,kw OR 'angle fracture':ti,ab,kw OR 'condylar fracture':ti,ab,kw) AND ('third molar':ti,ab,kw OR 'wisdom tooth':ti,ab,kw OR 'wisdom teeth':ti,ab,kw OR 'mandibular third molar':ti,ab,kw OR 'lower third molar':ti,ab,kw OR 'impacted tooth':ti,ab,kw OR 'impacted teeth':ti,ab,kw OR 'tooth impaction':ti,ab,kw) AND ('systematic review':ti,ab,kw OR 'meta-analysis':ti,ab,kw OR 'meta analysis':ti,ab,kw OR 'pooled analysis':ti,ab,kw OR 'systematic literature review':ti,ab,kw)) AND [english]/lim |

**Supplementary Table 2. Full-text screening results for the umbrella review**

| **No.** | **Database** | **Title of study** | **Citation** | **Included** | **Exclusion reason** |
| --- | --- | --- | --- | --- | --- |
| **1** | **Embase, PubMed, WoS** | Association between third molar and mandibular angle fracture: A systematic review and meta-analysis | Giovacchini F, Paradiso D, Bensi C, Belli S, Lomurno G, Tullio A. J Craniomaxillofac Surg. 2018;46(4):558-565. doi: 10.1016/j.jcms.2017.12.011 | Yes |  |
| **2** | **Embase, PubMed, WoS** | Does an Association Exist Between the Presence of Lower Third Molar and Mandibular Angle Fractures?: A Meta-Analysis | Ruela WDS, de Almeida VL, Lima-Rivera LM, Santos PL, Porporatti AL, de Freitas PHL, Paranhos LR. J Oral Maxillofac Surg. 2018;76(1):34-45. doi: 10.1016/j.joms.2017.06.008 | Yes |  |
| **3** | **Embase, PubMed, WoS** | How Is Third Molar Status Associated With the Occurrence of Mandibular Angle and Condyle Fractures? | Xu S, Huang JJ, Xiong Y, Tan YH. J Oral Maxillofac Surg. 2017;75(7):1476.e1-1476.e15. doi: 10.1016/j.joms.2017.03.021 | Yes |  |
| **4** | **Embase, PubMed, WoS** | Influence of third molars in mandibular fractures. Part 1: mandibular angle — a meta-analysis | Armond ACV, Martins CC, Glória JCR, Galvão EL, dos Santos CRR, Falci SGM. Int J Oral Maxillofac Surg. 2017;46(6):716-729. doi: 10.1016/j.ijom.2017.02.1264 | Yes |  |
| **5** | **Embase, PubMed, WoS** | Influence of third molars in mandibular fractures. Part 2: mandibular condyle — a meta-analysis | Armond ACV, Martins CC, Glória JCR, Galvão EL, dos Santos CRR, Falci SGM. Int J Oral Maxillofac Surg. 2017;46(6):730-739. doi: 10.1016/j.ijom.2017.02.1265 | Yes |  |
| **6** | **Embase, PubMed, WoS** | Do third molars weaken the mandibular angle? | Bezerra TP, Studart-Soares EC, Pita-Neto IC, Costa FW, Batista SH. Do third molars weaken the mandibular angle. Med Oral Patol Oral Cir Bucal. 2011 Aug 1;16(5):e657-63. | Yes |  |
| 7 | **Embase, PubMed, WoS** | The association of third molars with mandibular angle fractures: a meta-analysis | Hanson BP, Cummings P, Rivara FP, John MT. J Can Dent Assoc. 2004;70(1):39-43. PMID: 14709255 | Yes |  |
| 8 | **PubMed, WoS** | Mandibular third molar and angle fractures: a meta-analysis | Machete ML, Makwakwa LN, Khan A, Motloba DP. Mandibular third molar and angle fractures: a meta-analysis. South African Dental Journal. 2016 Nov 1;71(10):483-8. | Yes |  |
| 9 | **Embase, PubMed, WoS** | Effect of third molars in the line of mandibular angle fractures on postoperative complications: systematic review and meta-analysis | Fernandes IA, Souza GM, Silva de Rezende V, Al-Sharani HM, Douglas-de-Oliveira DW, Galvão EL, Falci SGM. Int J Oral Maxillofac Surg. 2020;49(4):471-482. doi: 10.1016/j.ijom.2019.09.017 | No | Postoperative complications |
| 10 | **Embase, PubMed, WoS** | Should Degree of Third Molar Eruption Influence Operative Management of Mandibular Angle Fractures? A Systematic Review | Kotha VS, de Ruiter BJ, Knudsen MG, Nicoleau M, Davidson EH. Craniomaxillofac Trauma Reconstr. 2022;15(4):379-386. doi: 10.1177/19433875211059330 | No | Operative management |
| 11 | **Embase, PubMed, WoS** | Late mandibular fracture occurring in the postoperative period after third molar removal: systematic review and analysis of 124 cases | Pires WR, Bonardi JP, Faverani LP, Momesso GAC, Muñoz XMJP, Silva AFM, Panzarini SR, Bassi APF, Ponzoni D. Int J Oral Maxillofac Surg. 2017;46(1):46-53. doi: 10.1016/j.ijom.2016.09.003 | NO | Other research question |
| **12** | **Embase, PubMed, WoS** | Bad splits in bilateral sagittal split osteotomy: systematic review and meta-analysis of reported risk factors | Steenen SA, van Wijk AJ, Becking AG. Int J Oral Maxillofac Surg. 2016;45(8):971-979. doi: 10.1016/j.ijom.2016.02.011 | No | Other research question |
| **13** | **Embase, PubMed** | Identifying the risk factors causing iatrogenic mandibular fractures associated with exodontia: a systemic meta-analysis of 200 cases from 1953 to 2015 | Joshi A, Goel M, Thorat A. Oral Maxillofac Surg. 2016;20(4):391-396. doi: 10.1007/s10006-016-0579-9 | No | Other research question |
| **14** | **Embase, PubMed** | Association between eruption state of the third molar and the occurrence of mandibular angle fractures | Rahimi-Nedjat RK, Sagheb K, Jacobs C, Walter C. Dent Traumatol. 2016;32(5):347-352. doi: 10.1111/edt.12260 | No | Not systematic review |
| **15** | **Embase** | Third molar along the line of fracture in mandible — a systematic review | Kirthika R, Visalakshi K, Balakrishnan R. Indian J Public Health Res Dev. 2019;10(12):2201-2205. doi: 10.37506/v10/i12/2019/ijphrd/192328 | No | Other research question |
| **16** | **Embase** | Transoperative mandibular angle fracture from third molar extraction, case report [Conference Abstract] | Cota R, Goto A, Dias L. Int J Oral Maxillofac Surg. 2019;48(Suppl):264-265. doi: 10.1016/j.ijom.2019.03.809 | No | Conference abstract |
| **17** | **Embase** | Mandibular angle fracture with presence of third molar included: Muay Thai professional fighter [Conference Abstract] | Cota RE, Goto A. Int J Oral Maxillofac Surg. 2019;48(Suppl):196. doi: 10.1016/j.ijom.2019.03.606 | No | Conference abstract |
| **18** | **PubMed, WoS** | Prevalence of intraoperative and postoperative iatrogenic mandibular fractures after lower third molar extraction: A systematic review | Guillaumet-Claure MA, Juiz-Camps AM, Gay-Escoda C. J Clin Exp Dent. 2022;14(1):e85-e94. doi: 10.4317/jced.58390 | No | Other research question |
| **19** | **PubMed, WoS** | Antibiotic prophylaxis in oral and maxillofacial surgery: a systematic review | Milic T, Raidoo P, Gebauer D. Br J Oral Maxillofac Surg. 2021;59(6):633-642. doi: 10.1016/j.bjoms.2020.09.020 | No | Other research question |
| 20 | **PubMed, WoS** | Unusual complications associated with third molar surgery: A systematic review | Brauer HU. Quintessence Int. 2009;40(7):565-572. PMID: 19626231 | No | Other research question |
| 21 | **PubMed, WoS** | Surgical management of bilateral mandibular angle fractures: A systematic review | Bullis SR, Goh EZ, Johnson NR. Br J Oral Maxillofac Surg. 2025;63(3):182-188. doi: 10.1016/j.bjoms.2025.01.004 | No | Other research question |
| **22** | **WoS** | Mandibular fractures associated with third molar extraction: A systematic review and meta-analysis | Crespo-Reinoso PA, Ayora KAO. J Oral Maxillofac Surg Med Pathol. 2026;38(1):11-15. doi: 10.1016/j.ajoms.2025.07.001 | No | Other research question |
| **23** | **WoS** | Does Prophylactic Removal of Mandibular Third Molar Affect Outcomes and Postoperative Complications in Bilateral Sagittal Split Osteotomy in Young Adults? | Sakhare PV, Gondhalekar R, et al. J Sci Soc. 2025;52(4):317-325. doi: 10.4103/jss.jss_91_24 | No | Other research question |
| **24** | **WoS** | The effectiveness of photobiomodulation therapy on inferior alveolar nerve injury: A systematic review and META-analysis | Ma Y, Yang M, Chen X, Qu W, Qu X, He P. PLoS One. 2023;18(8):e0287833. doi: 10.1371/journal.pone.0287833 | No | Other research question |
| **25** | **WoS** | Fractures of the mandibular condyle: A review of 466 cases. Literature review, reflections on treatment and proposals | Zachariades N, Mezitis M, et al. J Craniomaxillofac Surg. 2006;34(7):421-432. doi: 10.1016/j.jcms.2006.07.859 | No | Other research question |
| **26** | **Embase, PubMed, WoS** | A review on the classification of mandibulectomy defects and suggested criteria for a universal description | Jagtiani K, Gurav S, Singh G, Dholam K. J Prosthet Dent. 2024;132(1):270-277. doi: 10.1016/j.prosdent.2022.06.001 | No | Other research question |
| **27** | **PubMed** | Kinesiologic taping reduces morbidity after oral and maxillofacial surgery: a pooled analysis | Ristow O, Pautke C, Kehl V, Koerdt S, Hahnefeld L, Hohlweg-Majert B. Physiother Theory Pract. 2014;30(6):390-398. doi: 10.3109/09593985.2014.891068 | No | Other research question |

**Supplementary Table 3. Corrected Covered Area (CCA) Overlap Analysis**

*CCA = OC / ((k − 1) × N), where OC = total pairwise overlapping citations, k = number of meta-analyses, N = number of unique primary studies. Thresholds: <5% Slight; 5–10% Moderate; 10–15% High; >15% Very High.*

| **Outcome Category** | **k** | **Total Citations** | Unique studies | **OC** | **CCA** | **Interpretation** |
| --- | --- | --- | --- | --- | --- | --- |
| Mandibular angle fracture | 6 | 94 | 40 | 107 | 0.535 | Very High |
| Mandibular condylar fracture | 2 | 26 | 23 | 3 | 0.130 | High |
| **Overall** | **8^*^** | **120** | **42** | **178** | **0.605** | **Very High** |

** Xu et al. counted in both angle and condylar fracture categories. Overall CCA pools all eight meta-analyses (N = 42 unique primary studies across both outcome categories).*

**Supplementary Table 4. Primary study overlap matrix**

*✓ = study included in that meta-analysis. Blue = angle fracture meta-analyses; green = condyle fracture meta-analyses. Yellow row total (n) = study appears in ≥4 meta-analyses. Italicized study names = study identified in included meta-analyses but excluded from the present synthesis (see Supplementary Table 5 for reasons). Column totals = number of primary studies per meta-analysis.*

| **Primary Study** | **Angle Fracture Meta-Analyses** | | | | | | **Condyle Fracture Meta-Analyses** | | **n** |
| --- | --- | --- | --- | --- | --- | --- | --- | --- | --- |
|  | **Armond et al.** | **Bezerra et al.** | **Giovacchini et al.** | **Hanson et al.** | **Motloba et al.** | **Ruela et al.** | **Armond et al.** | **Xu et al.** | **Total** |
| *Abbasi et al. 2012* | **✓** |  |  |  |  |  |  |  | 1 |
| Antic et al. 2016 |  |  | **✓** |  |  |  |  | **✓** | 2 |
| Cho et al. 2006 | **✓** |  |  |  |  |  |  |  | 1 |
| Choi et al. 2011 | **✓** |  |  |  |  | **✓** | **✓** |  | 3 |
| Duan & Zhang 2008 | **✓** |  |  |  | **✓** | **✓** | **✓** |  | **4** |
| Fuselier et al. 2002 | **✓** | **✓** | **✓** | **✓** |  | **✓** |  | **✓** | **6** |
| Gaddipati et al. 2014 | **✓** |  |  |  | **✓** |  | **✓** | **✓** | **4** |
| Halmos et al. 2004 | **✓** | **✓** | **✓** |  | **✓** |  |  | **✓** | **5** |
| Iida et al. 2004 | **✓** |  |  |  |  | **✓** | **✓** |  | 3 |
| Iida et al. 2005 | **✓** | **✓** |  |  | **✓** | **✓** |  | **✓** | **5** |
| *Inaoka et al. 2009* | **✓** |  |  |  | **✓** |  | **✓** |  | 3 |
| Kasamatsu et al. 2003 | **✓** |  |  |  |  |  |  | **✓** | 2 |
| Kim 2004 | **✓** |  |  |  |  |  |  |  | 1 |
| Lee & Dodson 2000 | **✓** | **✓** | **✓** | **✓** | **✓** | **✓** |  | **✓** | **7** |
| Lee et al. 2012 | **✓** |  |  |  |  |  | **✓** |  | 2 |
| Luria & Campbell 2013 |  |  |  |  |  |  |  | **✓** | 1 |
| Ma'aita & Alwrikat 2000 | **✓** | **✓** | **✓** | **✓** | **✓** | **✓** |  |  | **6** |
| Mah et al. 2015 | **✓** |  |  |  | **✓** |  | **✓** |  | 3 |
| *Meisami et al. 2002* | **✓** | **✓** |  | **✓** |  |  |  | **✓** | **4** |
| *Metin et al. 2007* |  |  |  |  |  | **✓** |  |  | 1 |
| Naghipur et al. 2014 | **✓** |  |  |  | **✓** | **✓** | **✓** | **✓** | **5** |
| Oh et al. 2006 |  |  |  |  |  |  | **✓** |  | 1 |
| Park et al. 2009 | **✓** |  |  |  |  |  |  |  | 1 |
| Patil 2012 | **✓** |  |  |  | **✓** |  | **✓** |  | 3 |
| Rahimi-Nedjat et al. 2016 | **✓** |  |  |  |  |  |  |  | 1 |
| Rajandram et al. 2013 | **✓** |  |  |  | **✓** |  |  |  | 2 |
| Rajkumar et al. 2009 | **✓** |  |  |  | **✓** | **✓** |  |  | 3 |
| *Revanth Kumar et al. 2015* | **✓** |  |  |  |  | **✓** | **✓** |  | 3 |
| Safdar & Meechan 1995 | **✓** | **✓** |  |  | **✓** |  |  |  | 3 |
| *Schwimmer et al. 1983* |  | **✓** |  |  |  |  |  |  | 1 |
| Subhashraj 2009 | **✓** |  | **✓** |  | **✓** | **✓** |  |  | **4** |
| *Syed et al. 2015* |  |  |  |  | **✓** |  |  |  | 1 |
| Tankersly & Abubaker 1995 |  |  |  | **✓** |  |  |  |  | 1 |
| Tevepaugh & Dodson 1995 | **✓** | **✓** |  |  | **✓** | **✓** |  | **✓** | **5** |
| Thangavelu et al. 2010 | **✓** |  | **✓** |  | **✓** | **✓** | **✓** | **✓** | **6** |
| Ugboko et al. 2000 | **✓** | **✓** |  | **✓** | **✓** | **✓** |  | **✓** | **6** |
| *Yadav et al. 2013* | **✓** |  |  |  |  |  |  |  | 1 |
| *Yaltirik et al. 2002* | **✓** |  |  |  |  |  |  |  | 1 |
| Yamada et al. 1998 | **✓** | **✓** |  |  |  | **✓** |  |  | 3 |
| *Yu et al. 2011* | **✓** |  |  |  | **✓** |  |  |  | 2 |
| Zhu et al. 2005 | **✓** |  |  |  |  |  | **✓** |  | 2 |
| **Total studies per MA** | **35** | **11** | **7** | **6** | **19** | **16** | **13** | **13** | **41** |

**Supplementary Table 5. Excluded primary studies and reasons for exclusion (n=9)**

| **Author** | **Year** | **Country** | **N** | **Outcome** | **Reason for Exclusion** |
| --- | --- | --- | --- | --- | --- |
| **No fracture data (n = 1)** | | | | | |
| Syed et al. | 2013 | Saudi Arabia | NR | _ | No fracture data |
| **Incompatible Study Design (n = 7)** | | | | | |
| Yaltirik et al. | 2002 | Turkey | 100 | Angle | Incompatible design: compares unerupted vs erupted M3 only; no absent-M3 control group; p = 0.99 (NS) |
| Abbasi et al. | 2012 | Pakistan | 102 | Angle | Incompatible design: all patients had angle fractures; compares unerupted vs erupted only |
| Yadav et al. | 2013 | India | 289 | Angle | Incompatible design: cases only (all 289 had angle fractures); no comparator group |
| Revanth Kumar et al. | 2015 | India | 64 | Both | Incompatible design: compares impacted vs erupted M3; no absent-M3 data |
| Schwimmer et al. | 1983 | USA | NR | Angle | Incompatible design: restricted to contact sports injuries only; no absent-M3 control group |
| Metin et al. | 2007 | Türkiye | 41 | Angle | Incompatible design: compares impacted vs erupted M3; no absent-M3 data |
| Inaoka et al. | 2009 | Brazil | NR | Angle | Incompatible design: compares impacted vs erupted M3; no absent-M3 data |
| **No full text available (n = 1)** | | | | | |
| Yu et al. | 2011 | South Korea | 134 | Angle | No full text available |

**Supplementary Table 6. Quality assessment of the primary studies by Newcastle-Ottawa Scale**

| **Studies** | **Selection (max 4)** | | | | **Comparability (max 2)** | | | **Exposure / Outcome (max 3)** | | | | **Total (0–9)** | **NOS Quality** |
| --- | --- | --- | --- | --- | --- | --- | --- | --- | --- | --- | --- | --- | --- |
|  | **Case def./ Rep. of sample/ Rep. of exposed** | **Rep. of cases/ Sample size/ Sel. of non-exposed** | **Sel. of controls/ Asc. of exposure (0–2 CS)** | **Def. of controls/ –/ Outcome not present** | **Sub- total** | **Control for confounders (0–2)** | **Sub- total** | **Asc. of exp./ Assessment of outcome (0–2 CS)** | **Same method/ Stat. test/ Follow-up long enough** | **Non-response/ –/ Adequacy of follow-up** | **Sub- total** |  |  |
|  | **CC / CS / Cohort** | | | | **CC / CS / Cohort** | | | **CC / CS / Cohort** | | | |  |  |
| Tevepaugh & Dodson 1995 [1] | 1 | 1 | 1 | 0 | **3** | 0 | **0** | 1 | 1 | 1 | **3** | **6** | Moderate |
| Safdar & Meechan 1995 [2] | 1 | 0 | 1 | 1 | **3** | 0 | **0** | 1 | 1 | 1 | **3** | **6** | Moderate |
| Tankersly & Abubaker 1995 † [3] | — | — | — | — | — | — | — | — | — | — | — | **—** | Low † |
| Yamada et al. 1998 [4] | 1 | 0 | 1 | 0 | **2** | 0 | **0** | 1 | 1 | 1 | **3** | **5** | Low |
| Lee & Dodson 2000 [5] | 1 | 1 | 0 | 0 | **2** | 1 | **1** | 1 | 1 | 1 | **3** | **6** | Moderate |
| Ugboko et al. 2000 [6] | 1 | 0 | 1 | 0 | **2** | 0 | **0** | 1 | 1 | 1 | **3** | **5** | Moderate |
| Ma'aita & Alwrikat 2000 [7] | 1 | 1 | 1 | 1 | **4** | 1 | **1** | 1 | 1 | 1 | **3** | **8** | Moderate |
| Fuselier et al. 2002 [8] | 1 | 0 | 1 | 0 | **2** | 0 | **0** | 1 | 1 | 1 | **3** | **5** | Moderate |
| Kasamatsu et al. 2003 [9] | 1 | 0 | 1 | 0 | **2** | 0 | **0** | 1 | 1 | 1 | **3** | **5** | Moderate |
| Kim 2004 [10] | 1 | 0 | 1 | 0 | **2** | 1 | **1** | 1 | 1 | 1 | **3** | **6** | Moderate |
| Halmos et al. 2004 [11] | 1 | 1 | 1 | 0 | **3** | 2 | **2** | 1 | 1 | 1 | **3** | **8** | High |
| Iida et al. 2004 [12] | 1 | 0 | 1 | 0 | **2** | 1 | **1** | 1 | 1 | 1 | **3** | **6** | Moderate |
| Iida et al. 2005 [13] | 1 | 1 | 1 | 0 | **3** | 0 | **0** | 1 | 1 | 1 | **3** | **6** | Moderate |
| Zhu et al. 2005 [14] | 1 | 1 | 1 | 0 | **3** | 0 | **0** | 1 | 1 | 1 | **3** | **6** | Low |
| Cho et al. 2006 [15] | 1 | 0 | 1 | 0 | **2** | 1 | **1** | 1 | 1 | 1 | **3** | **6** | Moderate |
| Duan & Zhang 2008 [16] | 1 | 1 | 2 | 0 | **4** | 0 | **0** | 2 | 1 | 0 | **3** | **7** | High |
| Park et al. 2009 [17] | 1 | 0 | 1 | 0 | **2** | 1 | **1** | 1 | 1 | 1 | **3** | **6** | Moderate |
| Rajkumar et al. 2009 [18] | 1 | 0 | 1 | 0 | **2** | 0 | **0** | 1 | 1 | 1 | **3** | **5** | Low |
| Subhashraj 2009 [19] | 1 | 1 | 1 | 0 | **3** | 2 | **2** | 1 | 1 | 1 | **3** | **8** | High |
| Thangavelu et al. 2010 [20] | 1 | 0 | 1 | 0 | **2** | 1 | **1** | 1 | 1 | 1 | **3** | **6** | Moderate |
| Choi et al. 2011 [21] | 1 | 1 | 2 | 0 | **4** | 1 | **1** | 2 | 1 | 0 | **3** | **8** | High |
| Lee et al. 2012 [22] | 0 | 0 | 2 | 0 | **2** | 0 | **0** | 2 | 1 | 0 | **3** | **5** | Moderate |
| Patil 2012 [23] | 1 | 1 | 2 | 0 | **4** | 0 | **0** | 2 | 1 | 0 | **3** | **7** | High |
| Rajandram et al. 2013 [24] | 1 | 1 | 1 | 1 | **4** | 0 | **0** | 1 | 1 | 1 | **3** | **7** | High |
| Gaddipati et al. 2014 [25] | 1 | 1 | 2 | 0 | **4** | 0 | **0** | 2 | 1 | 0 | **3** | **7** | High |
| Naghipur et al. 2014 [26] | 1 | 1 | 1 | 0 | **3** | 1 | **1** | 1 | 1 | 1 | **3** | **7** | High |
| Mah et al. 2015 [27] | 1 | 1 | 2 | 0 | **4** | 1 | **1** | 2 | 1 | 0 | **3** | **8** | High |
| Rahimi-Nedjat et al. 2016 [28] | 1 | 0 | 1 | 0 | **2** | 1 | **1** | 1 | 1 | 1 | **3** | **6** | Moderate |
| Oh et al. 2006 [29] | 1 | 0 | 1 | 0 | **2** | 0 | **0** | 1 | 1 | 1 | **3** | **5** | Low |
| Luria & Campbell 2013 [30] | 1 | 1 | 1 | 0 | **3** | 0 | **0** | 1 | 1 | 1 | **3** | **6** | Moderate |
| Antic et al. 2016 [31] | 1 | 1 | 1 | 1 | **4** | 0 | **0** | 1 | 1 | 1 | **3** | **7** | Moderate |
| Nogami et al. 2018 [32] | 1 | 1 | 1 | 0 | **3** | 0 | **0** | 1 | 1 | 1 | **3** | **6** | Moderate |
| Mehra et al. 2019 [33] | 1 | 1 | 1 | 0 | **3** | 0 | **0** | 1 | 1 | 1 | **3** | **6** | Moderate |
| Lee Y et al. 2019 [34] | 1 | 1 | 1 | 0 | **3** | 1 | **1** | 1 | 1 | 1 | **3** | **7** | High |
| Soós et al. 2020 [31] | 1 | 1 | 1 | 1 | **4** | 1 | **1** | 1 | 1 | 0 | **2** | **7** | High |
| Brucoli et al. 2020 [36] | 1 | 1 | 1 | 0 | **3** | 0 | **0** | 1 | 1 | 1 | **3** | **6** | Moderate |
| Al-Sharani et al. 2021 [35] | 1 | 0 | 1 | 0 | **2** | 1 | **1** | 1 | 1 | 0 | **2** | **5** | Low |
| Venkatachalam et al. 2022 [36] | 1 | 0 | 1 | 0 | **2** | 0 | **0** | 1 | 1 | 1 | **3** | **5** | Low |
| Mu et al. 2024 [37] | 1 | 1 | 1 | 0 | **3** | 0 | **0** | 1 | 1 | 1 | **3** | **6** | Moderate |
| Meisami et al. 2002 [38] | 1 | 1 | 1 | 0 | **3** | 1 | **1** | 1 | 1 | 1 | **3** | **7** | High |
| Rozenbilds et al. 2026 [39] | 1 | 1 | 1 | 0 | **3** | 0 | **0** | 1 | 1 | 1 | **3** | **6** | Moderate |
| Al-Harbawee et al. 2021 [40] | 1 | 1 | 1 | 0 | **3** | 1 | **1** | 1 | 1 | 1 | **3** | **7** | High |

NOS = Newcastle–Ottawa Scale. Sub-item scores are integer star counts. CC = case–control study design: S1 = Case definition; S2 = Representativeness of cases; S3 = Selection of controls; S4 = Definition of controls; EO1 = Ascertainment of exposure; EO2 = Same method of ascertainment; EO3 = Non-response rate. CS = cross-sectional study design: S1 = Representativeness of sample; S2 = Sample size; S3 = Ascertainment of exposure (max 2 stars); S4 = not applicable (shown as 0); EO1 = Assessment of outcome (max 2 stars); EO2 = Statistical test; EO3 = not applicable (shown as 0). Cohort = cohort study design (Xu): S1 = Representativeness of exposed cohort; S2 = Selection of non-exposed cohort; S3 = Ascertainment of exposure; S4 = Outcome not present at start of study; EO1 = Assessment of outcome; EO2 = Follow-up long enough for outcomes to occur; EO3 = Adequacy of follow-up. Comparability (max 2): control for the most important factor and any additional factor. Quality thresholds: Low ≤ 5; Moderate 6; High ≥ 7.

**Supplementary Table 7. Leave-one-out sensitivity analysis for mandibular angle fracture measured by RR**

| **No.** | **Study Omitted** | **Pooled RR** | **95% CI** | **Direction of Change** |
| --- | --- | --- | --- | --- |
| **—** | **Overall (all 21 studies included)** | **2.18** | **1.66–2.87** | — |
| 1 | Mah et al. 2015 | **2.08** | 1.60–2.71 | ↓ Decreased by 0.10 |
| 2 | Duan & Zhang 2008 | **2.24** | 1.66–3.01 | ↑ Increased by 0.05 |
| 3 | Fuselier et al. 2002 | **2.19** | 1.64–2.94 | No meaningful change |
| 4 | Gaddipati et al. 2014 | **2.04** | 1.67–2.50 | ↓ Decreased by 0.14 |
| 5 | Halmos et al. 2004 | **2.13** | 1.60–2.84 | No meaningful change |
| 6 | Iida et al. 2005 | **2.19** | 1.64–2.92 | No meaningful change |
| 7 | Kasamatsu et al. 2003 | **2.15** | 1.62–2.85 | No meaningful change |
| 8 | Lee & Dodson 2000 | **2.20** | 1.65–2.94 | No meaningful change |
| 9 | Ma'aita & Alwrikat 2000 | **2.18** | 1.63–2.92 | No meaningful change |
| 10 | Meisami et al. 2002 | **2.16** | 1.62–2.88 | No meaningful change |
| 11 | Naghipur et al. 2014 | **2.21** | 1.65–2.96 | No meaningful change |
| 12 | Patil 2012 | **2.29** | 1.72–3.05 | ↑ Increased by 0.10 |
| 13 | Rajandram et al. 2013 | **2.12** | 1.60–2.81 | ↓ Decreased by 0.07 |
| 14 | Rajkumar et al. 2009 | **2.28** | 1.73–3.02 | ↑ Increased by 0.10 |
| 15 | Safdar & Meechan 1995 | **2.18** | 1.63–2.91 | No meaningful change |
| 16 | Subhashraj 2009 | **2.28** | 1.73–3.02 | ↑ Increased by 0.10 |
| 17 | Tevepaugh & Dodson 1995 | **2.16** | 1.63–2.86 | No meaningful change |
| 18 | Thangavelu et al. 2010 | **2.18** | 1.63–2.91 | No meaningful change |
| 19 | Ugboko et al. 2000 | **2.24** | 1.69–2.97 | ↑ Increased by 0.06 |
| 20 | Antic et al. 2016 | **2.21** | 1.65–2.96 | No meaningful change |
| 21 | Luria & Campbell 2013 | **2.19** | 1.64–2.93 | No meaningful change |

*RR, risk ratio; CI, confidence interval. ↑ = pooled RR increased relative to overall; ↓ = pooled RR decreased. Green = RR moved away from null; orange = RR moved toward null.*

**Supplementary Table 8. Assessment of the outcomes by GRADE framework**

| **Certainty assessment** | | | | | | | **№ of patients** | | **Effect** | | **Certainty** | **Importance** |
| --- | --- | --- | --- | --- | --- | --- | --- | --- | --- | --- | --- | --- |
| **№ of studies** | **Study design** | **Risk of bias** | **Inconsistency** | **Indirectness** | **Imprecision** | **Other considerations** | **With 3rd molar** | **Without 3rd molar** | **OR/RR (95% CI)** | **Absolute (95% CI)** | **Certainty** | **Importance** |
| ***Mandibular angle fracture – RR analysis (3rd molar present vs. absent)*** | | | | | | | | | | | |  |
| 21 | Observational studies (non-randomized) | Serious ^a^ | Serious ^b^ | Not serious | Not serious | Publication bias strongly suspected ^c^ | 11,045 | 3,528 | RR 2.18 (1.66–2.87) p < 0.0001 | — | **Very Low** | **CRITICAL** |
| ***Mandibular angle fracture – OR analysis (3rd molar present vs. absent)*** | | | | | | | | | | | |  |
| 20 | Observational studies (non-randomized) | Serious ^a^ | Not serious ^e^ | Not serious | Not serious | Strong association; all plausible residual confounding would reduce the demonstrated effect | — | — | OR 4.40 (3.29–5.88) p < 0.0001 | — | **Very Low** | **CRITICAL** |
| ***Mandibular condylar fracture – OR analysis (3rd molar present vs. absent)*** | | | | | | | | | | | |  |
| 20 | Observational studies (non-randomized) | Serious ^a^ | Serious ^b^ | Not serious | Serious ^d^ | All plausible residual confounding would reduce the demonstrated effect | 1,100 | 1,395 | OR 0.28 (0.19–0.43) p < 0.0001 | ~72% lower odds | **Very Low** | **CRITICAL** |

^a^ Risk of bias: Downgraded one level; all contributing studies were based on retrospective observational primary studies (case-control and retrospective cohort designs) with inherent selection bias.

^b^ Inconsistency: Downgraded one level; substantial heterogeneity detected (I² = 83–100%) reflecting differences in study populations, trauma mechanisms, geographic settings, and definitions of third molar status.

^c^ Publication bias: Downgraded one level; significant funnel plot asymmetry detected (Egger's test p = 0.02); overrepresentation of small positive studies likely.

^d^ Imprecision: Downgraded one level; limited number of contributing meta-analyses (n = 2) underpowering heterogeneity and precision estimates.

^e^ Inconsistency: Not downgraded; low heterogeneity across contributing meta-analyses (I² = 30.3%, p-heterogeneity = 0.15).

CI: Confidence interval; OR: Odds ratio; RR: Risk ratio.

**Supplementary Table 9. Leave-one-out sensitivity analysis for mandibular condylar fracture**

| **No.** | **Study Omitted** | **Pooled OR** | **95% CI** | **Direction of Change** |
| --- | --- | --- | --- | --- |
| **—** | **Overall (all 20 studies included)** | **0.28** | **0.18–0.42** | — |
| 1 | Iida et al. 2004 | **0.27** | 0.17–0.42 | No meaningful change |
| 2 | Choi et al. 2011 | **0.27** | 0.17–0.43 | No meaningful change |
| 3 | Zhu et al. 2005 | **0.28** | 0.18–0.44 | No meaningful change |
| 4 | Mah et al. 2015 | **0.27** | 0.17–0.43 | No meaningful change |
| 5 | Antic et al. 2016 | **0.26** | 0.17–0.40 | No meaningful change |
| 6 | Duan & Zhang 2008 | **0.26** | 0.17–0.41 | No meaningful change |
| 7 | Lee et al. 2012 | **0.28** | 0.18–0.43 | No meaningful change |
| 8 | Oh et al. 2006 | **0.26** | 0.17–0.41 | No meaningful change |
| 9 | Gaddipati et al. 2014 | **0.31** | 0.21–0.45 | No meaningful change |
| 10 | Patil 2012 | **0.28** | 0.18–0.44 | No meaningful change |
| 11 | Rozenbilds et al. 2026 | **0.26** | 0.17–0.40 | No meaningful change |
| 12 | Mehra et al. 2019 | **0.32** | 0.23–0.45 | ↑ Less protective by 0.04 |
| 13 | Al-Sharani et al. 2021 | **0.27** | 0.18–0.43 | No meaningful change |
| 14 | Lee Y et al. 2019 | **0.27** | 0.17–0.42 | No meaningful change |
| 15 | Venkatachalam et al. 2022 | **0.27** | 0.17–0.42 | No meaningful change |
| 16 | Mu et al. 2024 | **0.27** | 0.17–0.43 | No meaningful change |
| 17 | Soós et al. 2020 | **0.27** | 0.17–0.42 | No meaningful change |
| 18 | Nogami et al. 2018 | **0.29** | 0.19–0.45 | No meaningful change |
| 19 | Brucoli et al. 2020 | **0.29** | 0.19–0.45 | No meaningful change |
| 20 | Al-Harbawee et al. 2021 | **0.28** | 0.18–0.44 | No meaningful change |

*RR, risk ratio; CI, confidence interval. ↓ More protective = omitting this study makes the pooled RR smaller (stronger protective effect); ↑ Less protective = omitting this study moves RR closer to 1.0. Green = stronger protection; orange = weaker protection.*

**Supplementary Table 10. Meta-regression results**

| **Moderator** | **Outcome** | **Coefficient (log scale)** | **Exp(β)** | **SE** | **z** | **p-value** |
| --- | --- | --- | --- | --- | --- | --- |
| **Publication year** | | | | | | |
|  | Angle RR | 0.008 | 1.008 | 0.016 | 0.498 | 0.618 |
|  | Condyle OR | -0.008 | 0.992 | 0.025 | -0.310 | 0.756 |
| **Sample size (log-transformed)** | | | | | | |
|  | Angle RR | -0.186 | 0.831 | 0.123 | -1.507 | 0.132 |
|  | Condyle OR | 0.278 | 1.321 | 0.234 | 1.189 | 0.234 |
| **NOS score** | | | | | | |
|  | Angle RR | 0.109 | 1.115 | 0.096 | 1.134 | 0.257 |
|  | Condyle OR | -0.032 | 0.968 | 0.181 | -0.179 | 0.858 |
| **Fracture mechanism** | | | | | | |
|  | Angle RR | −0.076 | 0.927 | 0.110 | −0.694 | 0.488 |

tau² (DL) = 0.128 (angle fracture); 0.378 (condyle fracture). RR = risk ratio; OR = odds ratio; SE = standard error; NOS = Newcastle–Ottawa Scale; DL = DerSimonian–Laird; RTA = road traffic accident; NR = not reported.

**Supplementary Table 11. Summary of residual heterogeneity**

| **Outcome** | **Moderator** | **tau²** | **Residual Q** | **p (residual)** |
| --- | --- | --- | --- | --- |
| Angle RR (k=21) | Publication year | 0.154 | 35.90 | 0.011 |
|  | log(Sample size) | 0.154 | 33.88 | 0.019 |
|  | NOS score | 0.154 | 34.87 | 0.014 |
|  | Fracture mechanism | NR | NR | NR |
| Condyle OR (k=20) | Publication year | 0.417 | 28.96 | 0.049 |
|  | log(Sample size) | 0.417 | 27.64 | 0.068 |
|  | NOS score | 0.417 | 29.02 | 0.048 |

Bold p-values <0.05 indicate significant residual heterogeneity after accounting for the moderator. tau² = between-study variance; Q = Cochran’s Q statistic.

**Supplementary Figures:**

Supplementary Figure 1. Forest plot of trim and fill analysis of the risk ratio for mandibular angle fracture in the presence of third molars.

Supplementary Figure 2. Forest plot of the risk ratio of mandibular angle fracture in the presence of third molars: comparison between large vs. sample size studies

Supplementary Figure 3. Forest plot of the risk ratio of mandibular angle fracture in the presence of third molars stratified by study quality.

Supplementary Figure 4. Forest plot of the risk ratio of mandibular angle fracture in the presence of third molars stratified by time periods.

Supplementary Figure 5. Forest plot of the risk ratio of mandibular angle fracture in the presence of third molars stratified by fracture cause. NR: not reported

Supplementary Figure 6. Trim and fill forest plot analysis of the odds ratio for condylar angle fracture in the presence of third molars.

Supplementary Figure 7. Forest plot of the risk ratio of mandibular condylar fracture in the presence of third molars.

Supplementary Figure 8. Forest plot of the odds ratio of condylar fracture in the presence of third molars stratified by sample size.

Supplementary Figure 9. Forest plot of the odds ratio of condylar fracture in the presence of third molars stratified by study quality.

Supplementary Figure 10. Forest plot of the odds ratio of condylar fracture in the presence of third molars stratified by time periods.

Supplementary Figure 11. Forest plot of the odds ratio of condylar fracture in the presence of third molars stratified by fracture cause.

Supplementary Figure 12. Forest plot of the odds ratio of angle fracture risk stratified according to Pell and Gregory classification for third molar impaction

Supplementary Figure 13. Forest plot of the odds ratio of condyle fracture risk stratified according to Pell and Gregory classification for third molar impaction

**References:**

1.	Tevepaugh DB, Dodson TB. Are mandibular third molars a risk factor for angle fractures?: A retrospective cohort study. J Oral Maxillofac Surg. 1995;53(6):646–9.

2.	Safdar N, Meechan JG. Relationship between fractures of the mandibular angle and the presence and state of eruption of the lower third molar. Oral Surg Oral Med Oral Pathol Oral Radiol Endodontology. 1995;79(6):680–4.

3.	Tankersly K, Abubaker AO, Laskin DM. THE RELATIONSHIP BETWEEN PRESENCE OF MANDIBULAR 3RD MOLARS AND MANDIBULAR ANGLE FRACTURES. In: JOURNAL OF DENTAL RESEARCH. AMER ASSOC DENTAL RESEARCH 1619 DUKE ST, ALEXANDRIA, VA 22314; 1995. p. 80–80.

4.	Yamada T, Sawaki Y, Tohnai I, Takeuchi M, Ueda M. A study of sports‐related mandibular angle fracture: relation to the position of the third molars. Scand J Med Sci Sports. 1998 Apr;8(2):116–9. doi:10.1111/j.1600-0838.1998.tb00178.x

5.	Lee JT, Dodson TB. The effect of mandibular third molar presence and position on the risk of an angle fracture. J Oral Maxillofac Surg. 2000;58(4):394–8.

6.	Ugboko VI, Oginni FO, Owotade FJ. An investigation into the relationship between mandibular third molars and angle fractures in Nigerians. Br J Oral Maxillofac Surg. 2000;38(5):427–9.

7.	Ma’aita J, Alwrikat A. Is the mandibular third molar a risk factor for mandibular angle fracture? Oral Surg Oral Med Oral Pathol Oral Radiol Endodontology. 2000;89(2):143–6.

8.	Fuselier JC, Ellis III EE, Dodson TB. Do mandibular third molars alter the risk of angle fracture? J Oral Maxillofac Surg. 2002;60(5):514–8.

9.	Kasamatsu A, Watanabe T, Kanazawa H. Presence of the third molar as a risk factor in mandibular angle fractures. Asian J Oral Maxillofac Surg. 2003;15(3):176–80.

10.	Kim HK. Relationships between mandibular angle fracture and state of the lower third molar. J Korean Assoc Oral Maxillofac Surg. 2004;530–5.

11.	Halmos DR, Ellis III E, Dodson TB. Mandibular third molars and angle fractures. J Oral Maxillofac Surg. 2004;62(9):1076–81.

12.	Iida S, Nomura K, Okura M, Kogo M. Influence of the incompletely erupted lower third molar on mandibular angle and condylar fractures. J Trauma Acute Care Surg. 2004;57(3):613–7.

13.	Iida S, Hassfeld S, Reuther T, Nomura K, Mühling J. Relationship between the risk of mandibular angle fractures and the status of incompletely erupted mandibular third molars. J Cranio-Maxillofac Surg. 2005;33(3):158–63.

14.	Zhu SJ, Choi BH, Kim HJ, Park WS, Huh JY, Jung JH, et al. Relationship between the presence of unerupted mandibular third molars and fractures of the mandibular condyle. Int J Oral Maxillofac Surg. 2005;34(4):382–5.

15.	Cho SP, Lee JH, Kim CH. The influence of mandibular third molar on mandibular angle fracture. J Korean Assoc Maxillofac Plast Reconstr Surg. 2006;49–57.

16.	Duan DH, Zhang Y. Does the presence of mandibular third molars increase the risk of angle fracture and simultaneously decrease the risk of condylar fracture? Int J Oral Maxillofac Surg. 2008;37(1):25–8.

17.	박선혜, 임대호, 신효근, 고승오, 최준영, 김성일, et al. A study of correlation between mandibular angle fracture and the mandibular third molar. J Korean Assoc Oral Maxillofac Surg. 2009;35(6):420–5.

18.	Rajkumar K, Ramen S, Chowdhury R, Chattopadhyay PK. Mandibular third molars as a risk factor for angle fractures: a retrospective study. J Maxillofac Oral Surg. 2009 Sep;8(3):237–40. doi:10.1007/s12663-009-0058-z

19.	Subhashraj K. A study on the impact of mandibular third molars on angle fractures. J Oral Maxillofac Surg. 2009;67(5):968–72.

20.	Thangavelu A, Yoganandha R, Vaidhyanathan A. Impact of impacted mandibular third molars in mandibular angle and condylar fractures. Int J Oral Maxillofac Surg. 2010;39(2):136–9.

21.	Choi BJ, Park S, Lee DW, Ohe JY, Kwon YD. Effect of lower third molars on the incidence of mandibular angle and condylar fractures. J Craniofac Surg. 2011;22(4):1521–5.

22.	Lee YJ, Song YJ, Hong SM, Chae SS, Kang HW, Choi DJ, et al. The impact of the presence and aspect of mandibular third molars to the mandibular angle and condyle fractures. J Korean Dent Assoc. 2012 Sep 30;50(9):566–73. doi:10.22974/jkda.2012.50.9.004

23.	Patil PM. Unerupted lower third molars and their influence on fractures of the mandibular angle and condyle. Br J Oral Maxillofac Surg. 2012;50(5):443–6.

24.	Rajandram RK, Nabil S, Shareif MS, Ishak I, Marhazlinda J, Nordin R, et al. Mandibular third molar and angle of mandible fractures: an unsolved clinical dilemma. Sains Malays [Internet]. 2013 [cited 2026 Jun 19];42(1). Available from: https://knova.um.edu.my/research_publications_2011_2015/3512/

25.	Gaddipati R, Ramisetty S, Vura N, Kanduri RR, Gunda VK. Impacted mandibular third molars and their influence on mandibular angle and condyle fractures–A retrospective study. J Cranio-Maxillofac Surg. 2014;42(7):1102–5.

26.	Naghipur S, Shah A, Elgazzar RF. Does the presence or position of lower third molars alter the risk of mandibular angle or condylar fractures? J Oral Maxillofac Surg. 2014;72(9):1766–72.

27.	Mah DH, Kim SG, Moon SY, Oh JS, You JS. Relationship between mandibular condyle and angle fractures and the presence of mandibular third molars. J Korean Assoc Oral Maxillofac Surg. 2015;41(1):3.

28.	Rahimi‐Nedjat RK, Sagheb K, Jacobs C, Walter C. Association between eruption state of the third molar and the occurrence of mandibular angle fractures. Dent Traumatol. 2016 Oct;32(5):347–52. doi:10.1111/edt.12260

29.	Oh JK, Cha DW, Kim CS. The influence of the presence and impacted state of mandibular third molars on the incidence of mandibular condyle fracture. J Korean Assoc Maxillofac Plast Reconstr Surg. 2006;28(6):565–9.

30.	Luria JS, Campbell JH. Fracture patterns associated with the presence of mandibular third molars. J Oral Maxillofac Surg. 2013;71(9):e100.

31.	Antic S, Milicic B, Jelovac DB, Djuric M. Impact of the lower third molar and injury mechanism on the risk of mandibular angle and condylar fractures. Dent Traumatol. 2016 Aug;32(4):286–95. doi:10.1111/edt.12259

32.	Nogami S, Yamauchi K, Bottini GB, Kouketsu A, Otake Y, Sai Y, et al. Do mandibular third molars play a role in fractures of the mandibular angle and condyle? J Craniofac Surg. 2018;29(7):e713–7.

33.	Mehra A, Anehosur V, Kumar N. Impacted Mandibular Third Molars and Their Influence on Mandibular Angle and Condyle Fractures. Craniomaxillofacial Trauma Reconstr. 2019 Dec;12(4):291–300. doi:10.1055/s-0039-1685459

34.	Lee Y, Kim J, Lee M, Shin D, Choi H. Relationship between mandible fractures and third molars. Arch Craniofacial Surg. 2019;20(6):376.

35.	Mohammed Al-Sharani H, Bin Z, Ahmed Mashrah M, Galvão EL, Ahmed Al-Moraissi E, Ali Al-Aroomi M, et al. The influence of wisdom tooth impaction and occlusal support on mandibular angle and condyle fractures. Sci Rep. 2021;11(1):8335.

36.	Venkatachalam V, Pandiarajan R. Does the Impacted Mandibular Third Molar Increase the Risk of Angle Fracture to Prevent the Incidence of Condylar Fracture?–A Retrospective Analysis. Ann Maxillofac Surg. 2022;12(2):185–9.

37.	Mu J, Wu Y, Wu C, Piao H, Jin B. Relationship between mandibular third molars and mandibular angle and condylar fractures. Med Oral Patol Oral Cir Bucal. 2024;29(5):e634.

38.	Meisami T, Sojat A, Sàndor GKB, Lawrence HP, Clokie CML. Impacted third molars and risk of angle fracture. Int J Oral Maxillofac Surg. 2002;31(2):140–4.

39.	Rozenbilds M, Chung L, Thomas M. Prevalence of angle and condyle fractures according to the presence or absence of the mandibular third molar in patients with surgically treated mandible fractures. Int J Oral Maxillofac Surg [Internet]. 2025 [cited 2026 Jun 29]. Available from: https://www.sciencedirect.com/science/article/pii/S0901502725014912

40.	Al-Harbawee A, Ahmed T, Ahmed S, Avery C, Fagiry R, Hamzah HA, et al. A retrospective analysis of the impaction status of mandibular third molars as a risk factor for fractures of angle or condylar region of the mandible. Adv Oral Maxillofac Surg. 2021;1:100018.
