# Supplementary material for: Role of third molars in mandibular fractures: a systematic umbrella review with updated meta-analysis
Source: Front Oral Health. 2026 Aug 17;7:1865229. doi: 10.3389/froh.2026.1865229 (PMC13530431; doi:10.3389/froh.2026.1865229)
Supplement: Supplementary file 2 [file Supplementaryfile2.docx]

Presence of mandibular third molars is associated with a higher observed risk of mandibular angle fractures and a lower observed risk of condylar fractures.
